# Multi-Omics Uncover Neonatal Cecal Cell Development Potentials

**DOI:** 10.3389/fcell.2022.840298

**Published:** 2022-07-15

**Authors:** Liang Chen, Qingshi Meng, Shen Li, Yue Jiang, Cong Zhang, Shanlong Tang, Ruqing Zhong, Xiangfang Tang, Sheng Zhang, Xiaohui Feng, Yong Zhao, Hongfu Zhang

**Affiliations:** ^1^ State Key Laboratory of Animal Nutrition, Institute of Animal Sciences, Chinese Academy of Agricultural Sciences, Beijing, China; ^2^ Proteomics and Metabolomics Facility, Cornell University, Ithaca, NY, United States

**Keywords:** cecum, development, single cell RNA-seq (transcriptomics), proteomics, metabolomics, microbiota

## Abstract

Although, the cecum plays vital roles in absorption of water, electrolytes, and other small molecules, and harbors trillions of commensal bacteria to shape large intestine immune functions, it is unknown the cecum development potentials at single cell level during the very crucial neonatal developmental period. Using singe cell RNA-seq and proteomics, we have characterized six major types of cecal cells: undifferentiated cells; immune cells (Ims); cecumocytes (CCs); goblet, Paneth like cells (PLCs), and enteroendocrine cells (EECs) with specific markers. CCs mature with a gradual decrease in proportion of cells; however, Ims develop with a continuing increase in proportion of cells. Meanwhile, goblet and EEC cells reduced in proportion of cells from do to d14 or d21; PLCs increased in proportion of cells from d0 to d7 then decreased at d14 and d21. The cells exhibit specific development and maturation trends controlled by transcriptional factors, ligand-receptor pairs, and other factors. As piglets grow, cecal content and mucosal microbial diversity increases dramatically with population of beneficial microbiota, such as *lactobacillus*. Moreover, cecal mucosal-associated and cecal content microbiota are positively correlated and both show significant correlation with different types of cecal cells and plasma metabolites. This is the first presentation of neonatal cecal cell development and maturation naturally at single cell level with transcript, protein, microbiota and metabolism perspectives. Furthermore, this study provides an important tool for the determination of novel interventions in cecal drug delivery and metabolism studies.

## 1 Introduction

The large intestine, cecum, and colon, has the main functions of reabsorption of water, electrolytes and other small molecules, and elimination of undigested foodstuffs. The cecum lacks the finger-like projections known as villi and has little or no intrinsic digestive function (Dabareiner and White, 1997; Williams et al., 2001; Mowat and Agace, 2014; Peterson and Artis, 2014). However, trillions of commensal bacteria inhabit the cecum and colon and play essential roles in health (Mowat and Agace, 2014). In single-stomached animals, the large intestine is the most important site of fermentation and production of volatile fatty acids and this has important consequence for the health of the host (Williams et al., 2001). Moreover, the cells in the large intestine interact with the commensal microflora to form a symbiotic environment. These cells possess a functional role in innate and adaptive mucosal immunity (Peterson and Artis, 2014; Parikh et al., 2019). Moreover, immune cells within the intestine support the microbial communities and thus reinforce barrier function (Peterson and Artis, 2014).

The pig is recognized as an appropriate experimental animal model for human nutrition investigations because the physiological similarities between man and pig (Labib et al., 2004). Recently, the pig cecum model has been used in metabolic studies of many compounds and colon targeting for drug delivery (Von Engelhardt and Rechkemmer, 1992; Labib et al., 2004). A number of publications have explored the cell types and sub-types in small intestine or colon using single cell RNA-seq (scRNA-seq) (Haber et al., 2017; Gao et al., 2018; Parikh et al., 2019). They characterized the small intestinal cell diversity, and how the cell populations are regulated under pathogenic conditions (Gao et al., 2018; Zhao et al., 2020). Moreover, they studied the intestinal cell development during the fetal stage (Gao et al., 2018). However, we do not know cell types and sub-types in cecum. Furthermore, it is currently unknown how cecal cell types and sub-types differentiate throughout the neonatal period, or the molecular interactions (ligand-receptor, transcriptional factors, etc.) of cell types or subtypes during this critical developmental window. As the cecum is important in health, this study was conducted to investigate the differentiation of cecal cell types and sub-types especially during the critical neonatal developmental window, and cecum developmental potentials by multi-omics analyses.

## 2 Materials and Methods

### 2.1 Piglets

All animal procedures used in this study were approved by the Animal Care and Use Committee of the Institute of Animal Sciences of the Chinese Academy of Agricultural Sciences. Twenty-five piglets, born at full-term from three pure line large white sows were used in current study. All piglets were maintained in the same heat preserving pigsty at 28°C and fed solely on maternal milk (no, antibiotics, immunizations, or additives). Piglet plasma, cecal mucosa, and cecal content were collected from 5 piglets at each time point: d0 (at birth; *n* = 5), d1 (1 d post-birth; *n* = 5), d7 (7 d post-birth; *n* = 5), d14 (14 d post-birth; *n* = 5), and d21 (21 d post-birth; *n* = 5). At each collection point, 3 samples of cecal mucosal tissue were taken: 1) cecal tissue was cut and fixed in 10% formaldehyde for processing into histochemical blocks; 2) cecal mucosa was washed thrice in PBS buffer, gently scraped, and the removed tissue was used for the isolation of single cells for scRNA-seq analysis; 3) cecal mucosa was washed thrice in PBS buffer, gently scraped, and the removed tissue was quickly frozen in liquid nitrogen, and kept in −80°C refrigerator for proteomics and Western blotting analyses (Supplementary Figure S1A; Meng et al., 2021; Zhang et al., 2020; Zhao et al., 2020).

### 2.2 Single Cecal Cell Isolation, Library Preparation and Sequencing, and Data Analysis

#### 2.2.1 Single Cell Isolation, Library Preparation, and Sequencing

Single-cell libraries were constructed using the 10x Genomics Chromium Single Cell 3′ Library and Gel Bead Kit v.2 (10 × Genomics Inc., Pleasanton, CA, United States; 1,20,237) according to the manufacturer’s instructions. Protocols for single-cell sample preparation, library construction, and sequencing were performed according to our previous reports (Zhao et al., 2020; Meng et al., 2021)**,** and that of Haber et al. (2017). In summary, piglet ceca sections were collected and washed with PBS (Meng et al., 2021). Tissues were then incubated in 20 mM EDTA-PBS, on ice, for 90 min with agitation every 30 min. After 90 min of incubation, the samples were vigorously agitated and the supernatant was transferred to a new tube. The samples were then incubated with new 20 mM EDTA-PBS on ice for 30 min, and the supernatant collected again. In this way, four different components were collected and then combined. After centrifugation at 300 *g* for 3 min, the individual cell pellets were collected and washed twice with PBS using the same centrifugation protocol. Cells were then trypsinized (Invitrogen) for 1 min at 37°C and single cells were collected using a 40 μm filter. Cells were further washed twice with a PBS solution supplemented with 0.04% bovine serum albumin (BSA, Sigma, St. Louis, MO, United States; A1933). Cell viability was investigated using trypan blue stain and a hemocytometer (Bio-Rad, Hercules, CA, United States; TC20), and it was about 95%. Five cecal samples from five piglet were taken individually, and cells from the five samples were mixed together at each time point. Finally, all cell were pooled together for each time point. A concentration of 1,000 cells/μl was created and subsequently loaded onto a single cell chip (one/group). Chromium 10x Single Cell System (10 × Genomics) was used with a Gel Bead in EMulsions (GEMs) system. After treatment, cells were sorted using barcodes and cDNA library was constructed. Sequencing was performed using an Illumina Novaseq 6,000 sequencer (Illumina, San Diego, CA, United States) with pair end 150 bp (PE150) reads.

#### 2.2.2 Single Sample Analysis and Aggregation

CellRanger software (https://www.10xgenomics.com/) was used for dataset processing, using the “--force-cells = 5,000” parameter. The porcine reference genome (https://www.ncbi.nlm.nih.gov/assembly/GCF_000003025.6/) was constructed using the “cellranger mkgtf” function. After analysis with CellRanger, the gene barcode matrix was processed using the Seurat single cell RNA seq analysis R package in Rstudio (v3.0) (Butler et al., 2018; Meng et al., 2021). Cells with <200 genes and genes expressed in <3 cells were removed to obtain high-quality datasets for downstream analysis. After normalization, 5 datasets (one from each time point) were merged using the Seurat RunMultiCCA function. The characterized cell clusters were reviewed under the Uniform Manifold Approximation and Projection (UMAP). Cell clusters were counted using the FindClusters function, and cell cluster markers were identified using the Seurat FindAllMarkers function.

*Sub-clustering, Gene Ontology enrichment analysis* as described in our previous and other studies (Butler et al., 2018; Meng et al., 2021). When all cell clusters in the piglet cecal samples had been characterized, cells were clustered again according to cell identity. The SubsetData function was used to obtain similar cell types for downstream analysis. After clustering was completed, cluster-specific marker genes were identified using the FindAllMarkers function and marker genes were used by Metascape (http://metascape.org) for enrichment analysis (Butler et al., 2018; Meng et al., 2021).

#### 2.2.3 Single-Cell Pseudo-Time Trajectory Analysis

Single-cell pseudo-time tracks (http://cole-trapnell-lab.github.io/monocle-release/tutorials/) (Trapnell et al., 2014; Qiu et al., 2017; Meng et al., 2021) was determined using Monocle 2. Monocle objects were created from Seurat objects using the newCellDataSet function implemented by Monocle with a lowerDetectionLimit of 0.5. Seurat was used to identify variable genes for ordering. The DDRTree method was used to construct dimensionality by regressing the number of UMIs. Root states were appropriated based on the identity information of Seurat cell. Branch-specific gene expression was calculated using the BEAM function in Monocle. The branched heatmap was further constructed using the “plot_genes_branched_heatmap” function.

#### 2.2.4 Single Cell Regulatory Network Analysis

To identify gene regulatory networks that are active during cecal cell development, we used SCENIC for regulatory network inference and clustering (https://github.com/aertslab/SCENIC); a method to infer genes from single-cell RNA-seq data for regulatory networks (Aibar et al., 2017; Zhao et al., 2020; Meng et al., 2021). During analysis of single-cell RNA-seq expression matrices, cell IDs and genes were placed in columns and rows, respectively. Subsequently, genes with UMI counts<100 in all samples and genes expressed in <1% of cells were removed using gene filtering. Co-expression substrates containing potential regulators were then inferred with GENIE3. Afterwards, based on DNA motif analysis, protential direct binding targets were identified using RcisTarget; the databases (mm 10) were used that scored motifs in the promoter of the genes (up to 500 bp upstream the TSS), and in the 10 kb around the TSS (+/−10 kb). Regulon activity in each cell was calculated using the AUCell algorithm and network activity was converted into ON/OFF (binary activity matrix) with default settings.

#### 2.2.5 RNA Velocity Analysis Using Velocyto

Using the previously described velocyto software package (Meng et al., 2021), RNA velocity was used to determine whether a differentiation relationship exists in neonatal cecal cells. Standard protocols were used to generate counts of un-spliced and spliced mRNA in piglet cecal cells using the velocyto CLI. RNA velocity was then determined in all types of cecal cells (‘all’), or specific types of cecal cell using a similar workflows and parameters. Subsequently, RNA velocity was calculated while assuming constant velocity and transition probability, and embedding shift was calculated based on the previously generated UMAP representation of the cecal dataset.

#### 2.2.6 Protein–Protein Network (Ligand-Receptor) Enrichment Analysis

(Vento-Tormo et al., 2018; Efremova et al., 2020; Meng et al., 2021) ***.*** CellPhoneDB analysis [CellPhoneDB Python package (1.1.0)] was used to determine how context-dependent crosstalk of differing cell types enabled physiological processes to proceed; CellPhoneDB is a public repository of curated receptors, ligands, and their interactions. Cell-cell interaction analysis was determined after inputting single-cell data from all cell types into CellPhoneDB. The abundant receptor-ligand interactions between two cell types were derived from the receptor expression of one cell type and a corresponding ligand expression by another cell type. We then identified the most relevant cell type-specific interactions between ligands and receptors. Consideration was given to those receptors and ligands expressed in >10% of cells in the corresponding subclusters. Pairwise comparisons were made between the selected cell types. We first randomly permuted the cluster labels of all cells 1,000 times to determine the average receptor and ligand expression levels of interacting clusters. As a result, a null distribution was generated for each receptor-ligand pair. By calculating the proportion of the means that were greater than the actual mean, a *p*-value for the likelihood of cell type specificity of the corresponding receptor-ligand complex was achieved. All biologically relevant interactions were then selected.

### 2.3 Proteomics Analysis

Cecal sample proteomics analysis was performed as reported in our earlier publications (Hou et al., 2020; Liu et al., 2020; Meng et al., 2021).

#### 2.3.1 Protein Extraction and Digestion

Cecal mucosa was homogenized in lysis buffer (containing: 100 mM Tris-HCl pH 8.5, 7 M Urea, 1% SDS, 5 mM TCEP, protease inhibitors cocktail) at RT. Protein concentrations were determined using the bicinchoninic assay (BCA), in which 50 ug of protein was reduced with 5 mM TECP at 56°C for 30 min, followed by alkylation with 20 mM iodoacetamide in the dark, at RT for 30 min. Proteins were then precipitated using methanol/chloroform. 4, 1, and 3 volumes of methanol, chloroform, and water, were added to the lysate respectively; after each solvent was added, vortexing was performed; and then performed a final centrifugation of 5,000 g for 5 min at RT. After removal of the supernatant, the precipitate was washed with cold methanol, and the samples were air dried. The precipitate was then resuspended in 100 ul of digestion buffer (100 mm TEAB buffer; pH 8.0), trypsin was added at 1:25 (w/w); and protein digestion was performed overnight at 37°C.

#### 2.3.2 TMTpro labeling

Two sets of TMTpro plex amine reactive reagents were used to label 30 samples (Hou et al., 2020; Liu et al., 2020; Meng et al., 2021). Channel 126 was used to label an equally proportioned sample as that found in the reference channel. Briefly, the reactive reagents were resuspended in 30 μl of anhydrous acetonitrile; these were added to each sample and mixed by vortexing. The reactions were run - at RT for 1 h, and then halted by the addition of 8 μl of 5% hydroxylamine for 15 min. Labeled samples were pooled, lyophilized, and resuspended in 20 μl of 0.1% formic acid and 2% acetonitrile in water. The peptides were then loaded onto a Waters XBridge C18 column (5 μm; 4.6 × 100 mm, 120 Å). Buffer A was ammonium formate in water (10 mM; pH 10) and buffer B was ammonium formate in acetonitrile (10 mM; pH 10). The following gradients were used to separate peptides: 0–3 min, 5% B; 3–40 min, 60% B; 40–48 min, 80% B; 48–52 min, 80% B; 52–53 min, 5% B; and 53–55 min, 5% B. The collected 44 fractions were dried in a SpeedVac, mixed into 11 fractions, and resuspended in 0.1% formic acid and 2% acetonitrile for subsequent nano LC-MS/MS analysis.

#### 2.3.3 LC-MS/MS

Nano LC-MS/MS analysis was performed using an Orbitrap Fusion Tribrid MS (Thermo Scientific, San Jose, CA, United States) equipped with a nanospray flexible ion source, and coupled with a Dionex UltiMate 3000 RSLC nano system (Thermo, Sunnyvale, CA, United States). Peptide samples (2 μl) were injected into the PepMap C18 columns (75 μm × 3 mm, 3 μm) at a rate of 6 μl/min for on-line enrichment, followed by separation with a PepMap C18 column (2 μm, 75 μm × 250 mm), using 0.1% formic acid as buffer A and 0.1% formic acid in 80% acetonitrile as buffer B at 300 nl/min. The peptides were eluted using the followed gradients: 0–5 min, 5–12% B; 5–65 min, 12%–38% B; 65–72 min, 38–95% B; 72–80 min, 95% B; 80–81 min, 95–5% B; and 81–95 min, 5% B.

The mass spectrometers were set-up to use electrospray ionization (2 kV) at 275°C in “Top Speed” mode. Orbitrap resolution was 120 000; tandem mass spectrometry (MS/MS) was 50 000. MS/MS spectra were acquired using a quadrupole isolation width of 1.6 m/z and an HCD normalized collision energy (NCE) of 38. Dynamic exclusion was set for 30 s using monoisotopic precursor selection.

#### 2.3.4 Data Processing

Raw data files were searched using MSFragger v.3.11 and Philosopher v.3.3.11 against the *Sus scrofa* protein database from the NCBI database (GCF_000003025.6_Sscrofa11.1). The mass tolerances for precursor and fragment ions were 10 ppm and 0.02 Da, respectively. Filtering of proteins and peptides was performed with a false discovery rate (FDR) of <1%. Enzyme parameters were limited to semi-tryptic peptides with a maximum miscleavage of 2. Carbamidomethyl (C) of the peptides was set as the fixed modification; the oxidation (M) and deamidated (NQ) of the protein N-terminus were set as variable modifications. The reported ion intensities were filtered using Physpher to R with “PDtoMSstatsTMTFormat ()” from the MSstatsTMT package.

### 2.4 Cecal mucosa and Content Microbiota Sequencing as Described in Our Previous Studies (Zhang et al., 2020; Meng et al., 2021)

#### 2.4.1 DNA Extraction

Total genomic DNA from cecal mucosa and contents was isolated using the E. Z.N.A. ® Stool DNA Kit (Omega Bio-tek Inc., United States), following the manufacturer’s instructions. DNA quantity and quality were analyzed using NanoDrop 2000 (Thermo Scientific, United States) and 1% agarose gel.

#### 2.4.2 Library Preparation and Sequencing

The V3-V4 region of the 16S rRNA gene was amplified using barcoded primers 338F (5′- ACT​CCT​ACG​GGA​GGC​AGC​AG-3′) and 806R (5′-GGACTACHVGGGTWTCTAAT-3′). PCR reactions (total 30 μl) included 15 μl PhusionR High-Fidelity PCR Master Mix (New England Biolabs), 0.2 mM primers, and 10 ng DNA. The thermal cycle performed initial denaturation at 98°C, followed by 30 cycles of 98°C for 10 s, 50°C for 30 s, 72°C for 30 s, and a final extension at 72°C for 5 min. PCR products were purified using the AxyPrep DNA Gel Extraction Kit (Axygen Biosciences, United States). Sequencing libraries were constructed with a NEB Next^®^ UltraTM DNA Library Prep Kit for Illumina (NEB, United States) following the manufacturer’s instructions; index codes were added. The library was then sequenced on an Illumina MiSeq 2,500 platform (Illumina, United States) and generated 300 bp paired-end reads at the Novo gene. FLASH (v.1.2.71) was used to merge paired-end reads. Tag quality was controlled in QIIME (v.1.7.02), and all chimeras were removed. The “Core Set” of the Greengenes database3 was used for classification, and sequences with >97% similarity were assigned to the same operational taxonomic units (OTUs).

#### 2.4.3 Analysis of Sequencing Data

OTU abundance information was normalized using a standard of sequence numbers corresponding to the sample with the least sequences. Alpha diversity indices were calculated using QIIME (v.1.7.0). PLS-DA was performed with R software (v.2.15.3).

### 2.5 Plasma metabolites Determined by LC-MS/MS as Described in Our Previous Studies (Zhang et al., 2020; Meng et al., 2021)

Piglet plasma was collected and kept at −80°C. The protein was removed from the plasma samples on ice before LC-MS/MS analysis using ACQUITY UPLC and AB Sciex Triple TOF 5600 (LC/MS) as described previously.

The condition for HPLC was: ACQUITY UPLC BEH C18 column (100 mm × 2.1 mm, 1.7 μm), solvent A [aqueous solution with 0.1% (v/v) formic acid], and solvent B [acetonitrile with 0.1% (v/v) formic acid] with a gradient program: 0–2 min, 5–20% B; 2–4 min, 20%–25% B; 4–9 min, 25–60% B; 9–17 min, 60–100% B; 17–19 min, 100% B; 19–19.1 min, 100–5% B; and 19.1–20.1 min, 5% B. The flow rate was set at 0.4 ml/min and 5 μl was injected. ESI was used in the mass spectrometry program. Progenesis QI v. 2.3 (Nonlinear Dynamics, Newcastle, United Kingdom) was applied to normalize the peaks. Data were characterized using the Human Metabolome Database (HMDB), Lipidmaps (v. 2.3), and METLIN software. In addition, the data were analyzed with SIMCA software (v. 14.0, Umetrics, Umea, Sweden) and KEGG database (http://www.genome.jp/KEGG/pathway.html) was applied for the pathway enrichment analysis (Zhang et al., 2020).

### 2.6 Histopathology Analysis

Cecal tissue segments were fixed in 10% neutral formalin, paraffin embedded, cut into 5 μm sections, and stained with hematoxylin and eosin (H&E) for histopathological analysis.

### 2.7 Immunofluorescent Staining (IHF)

The protocol for immunofluorescence staining was reported in our recent publications (Zhang et al., 2020; Zhao et al., 2020; Meng et al., 2021). Supplementary Table S1 listed the primary antibodies that were used. In brief, riefly, 5 μm thick tissue sections were rehydrated gradient, subjected to antigen retrieval, and first blocked with normal goat serum in TBS, followed by incubation (1:150 in TBS-1% BSA) with primary antibodies at 4°C overnight. Sections were washed (TBS-1‰ Tween 20, 10 min X3) and then incubated with a Cy3/FITC labeled goat anti-rabbit or donkey anti-goat secondary Abs (1:150 in TBS-1% BSA; Beyotime Institute of Biotechnology, Shanghai, P.R. China) at 37°C for 30 min. After three times washes with TBST and then counterstained with Hoechst 33,342. Stained sections were examined under a Nikon Eclipse TE2000-U fluorescence microscope (Nikon, Inc., Melville, NY), and the resulting fluorescence images were analyzed with ImageJ software.

### 2.8 Western Blotting

Western blotting analysis followed our previously reported protocols (Zhang et al., 2020; Zhao et al., 2020; Meng et al., 2021). Briefly, cecal mucosal tissue samples were lysed in RIPA buffer containing a protease inhibitor cocktail from Sangong Biotech, Ltd. (Shanghai, China). Protein concentration was determined using a BCA kit (Beyotime Institute of Biotechnology). Information regarding the primary antibodies used is given in Supplementary Table S1. Actin was used as the loading control. Secondary donkey anti-goat Abs (Cat no: A0181) was purchased from Beyotime Institute of Biotechnology, and goat anti-rabbit (Cat no: A24531) Abs were purchased from Novex® by Life Technologies (United States). Protein samples (50 μg/sample) were loaded onto 10% SDS polyacrylamide electrophoresis gels. The gels were transferred to a polyvinylidene fluoride (PVDF) membrane at 300 mA for 2 h at 4°C. Membranes were then blocked with 5% BSA for 1 h at RT, followed by 3 washes with 0.1‰ Tween-20 in TBS (TBST). Membranes were then incubated with primary Abs diluted to 1:500 in TBST with 1% BSA overnight at 4°C. Following a further 3 washes with TBST, the blots were, respectively, incubated with the HRP-labelled secondary goat anti-rabbit or donkey anti-goat Ab, for 1 h at RT. Secondary donkey anti-goat Ab (Cat no: A0181) was purchased from Beyotime Institute of Biotechnology, and goat anti-rabbit (Cat no: A24531) Abs were purchased from Novex® by Life Technologies. After three washes, the blots were imaged.

### 2.9 Statistical Analysis

For cecal mucosa or content microbiota data analysis, data that were not normally distributed following log transformation or that had un-equal variances were subjected to nonparametric analysis using the Kruskal–Wallis test within the NPAR1WAY procedure of SAS.

### 2.10 Data Availability

The 10x sequencing raw data are deposited in NCBI’s Gene Expression Omnibus under accession number: GSE163272. Proteomics data are deposited at the Integrated Proteome resources (https://www.iprox.org/) with the ID: IPX0002622002. The microbiota raw sequencing data generated in this study has been uploaded to the NCBI SRA database with the accession number PRJNA688810.

## 3 Results

### 3.1 Profile of Neonatal Porcine Cecal Development at the Single Cell Level

**FIGURE 1:**
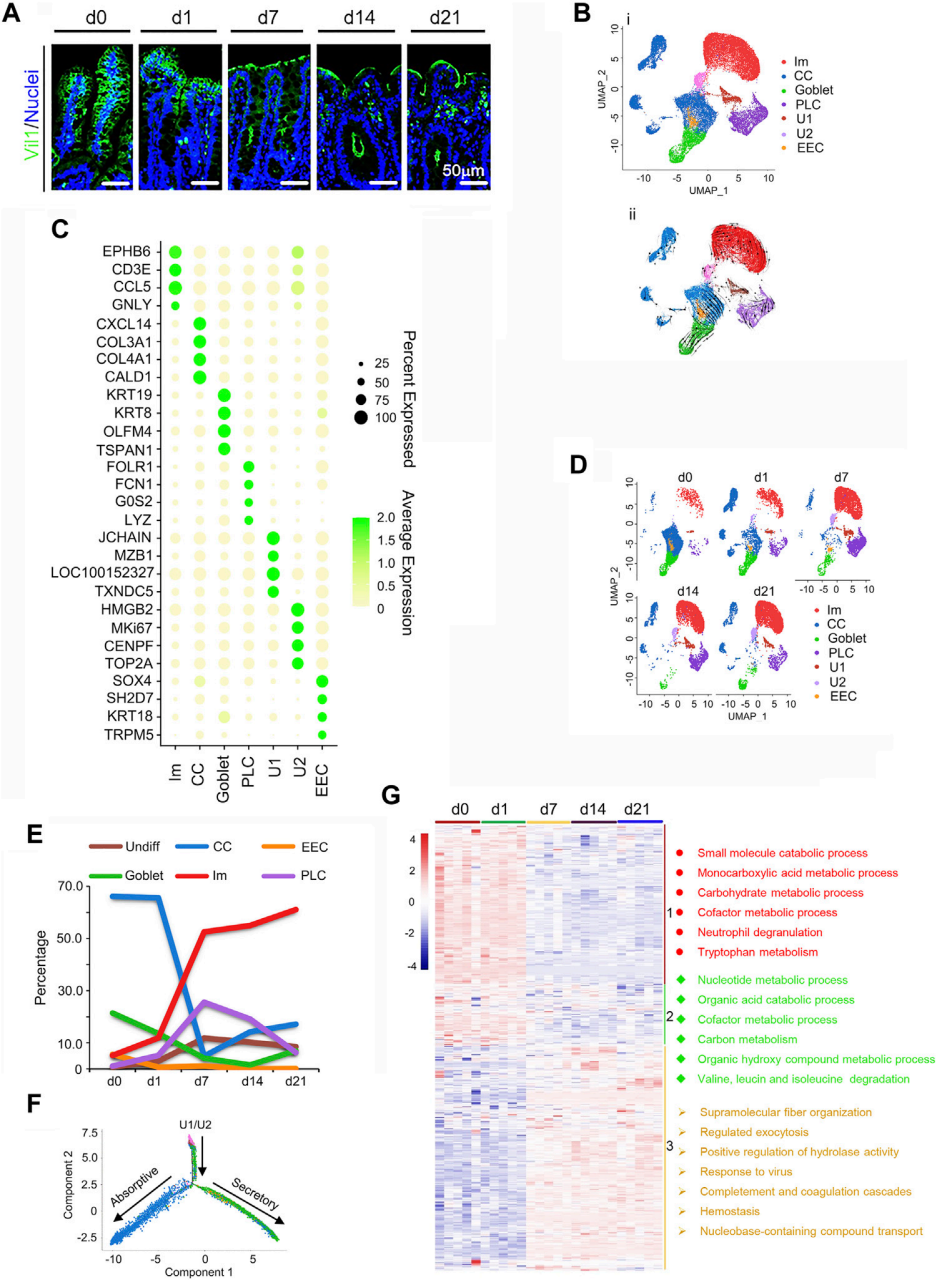
Single-cell profile of swine neonatal cecal epithelium. **(A)** Vil1 staining of the ceca at 5 time points in cecal samples. Scale bar: 50 μm. **(B)** Cecal cell type clusters (All the cells pooled together to show the major cell clusters for the five timepoints). (i). UMAP of 32 686 single cells (points), colored by cluster assignment (*n* = 5 piglets at each time point). (ii) RNA velocity vector projection on UMAP plot (The arrow indicated the developmental trend). **(C)** Heatmap of cluster marker genes, colored by relative gene expression. Dot size represents the fraction of cells per cluster. The color scale bar represented the average expression. **(D)** Cell population changes during development by UMAP analysis (from d0 to d21). **(E)** The proportion of cells in each cluster at each time point to show the trend of the relative percentage of cells from d0 to d21. **(F)** Differentiation pseudotime trajectory analysis of absorption and secretory cells (CCs, goblet, PLCs, and EECs) from undifferentiated cells (U1/U2). Predicted secretory-lineage cells and absorptive cells are from U1/U2. **(G)** Proteomics data of piglet cecal mucosa at 5 time points (five animal samples at each time point) with enriched functions for each group of proteins. Three clusters of proteins indicated by the different color bar on the right, and with the enriched main functional pathways.

In this study, we investigated the piglet cecal development during the neonatal window [from birth (d0) to 21 days of age (d21)] through scRNA-seq, proteomics, gut microbiota, and plasma metabolism (Supplementary Figure S1A; Study scheme). The piglet cecum developed gradually during its mucosal layer maturation (Figure 1A; Supplementary Figure S1B). At birth (d0), there were finger-like projections known as villi in the cecum similar as in the small intestine (Mowat and Agace, 2014). As the piglet grew, the projections became smaller and flatter to represent the cecal maturation lacking villi and a brush border showed by the vil1 staining (Figure 1A; Supplementary Figure S1C). A single-cell profiling was applied to create a map of cecal epithelia of the piglets at d0, d1, d7, d14, and d21 with 6,861, 6,451, 6,520, 6,415, and 6,439 cells (after quality control), respectively (Figures 1B–F; Supplementary Figure S1D–F; Supplementary Table S2). All the cells were combined together for further analysis and 6 clusters were partitioned by unsupervised graph clustering visualized by Uniform Manifold Approximation and Projection (UMAP; Figure 1B) as reported in early articles (Gao et al., 2018; Parikh et al., 2019; Zhao et al., 2020). This is the first study to cluster the cecum cells using scRNA-seq analysis using a method similar to Parikh et al. (2019).

The six clusters of cecal cells (Parikh et al., 2019) include undifferentiated cells [undifferentiated 1 cells (U1) and undifferentiated 2 cells (U2)], immune cells (Ims), cecal enterocyte (cecumocytes; CCs) (Parikh et al., 2019), goblet, Paneth like cells (PLCs), and enteroendocrine cells (EECs) with corresponding marker genes (Figure 1C; Supplementary Figure S1E, F; Supplementary Table S3). The timing of cell developmental potential was confirmed by RNA velocity analysis (Figure 1Cii) (La Manno et al., 2018; Joost et al., 2020).

Cecal development during the neonatal window was reflected by the proportion of different clusters of cells at different times (Figures 1D,E). Figure 1D showed the UMAP maps for the samples in different time points with each cell type. Figure 1E presented the percentage of each type of cells at each time point. The change of the number of the cells during the time was presented in Supplementary Figure S1G. At d0, CCs were most abundant, followed by goblet, EECs, Ims, PLCs, and U1/U2. However, with advancing age, CCs decreased dramatically to a minimum at d7, then increased at d14 and d21 (Figures 1D,E). On the other hand, Ims increased dramatically from d0 to d7, then continued to rise in proportion at d14 and d21 (Figures 1D,E). Meanwhile, U1/U2 gradually increased throughout the period (Figures 1D,E). At the same time, goblet cells sharply reduced from d0 to d14, and EECs gradually reduced from d0 to d21; while PLCs increased from d0 to d7, then gradually decreased till d21. At d21, Ims were most common (61.08%), followed by CCs (17.11%), U1/U2 (8.50%), goblet (6.97%), PLCs (6.27%), and EECs (0.06%; Figures 1D,E). All cell types approached mature cecum development at d21 (Mowat and Agace, 2014; Peterson and Artis, 2014). U1/U2, CCs, goblet, PLCs, and EECs were isolated and a bifurcating trajectory (pseudotime analysis) was found for these cell clusters, arising from U1/U2, then separating to secretory and absorptive lineages (Figure 1F) (Parikh et al., 2019).

Proteomics of the cecum was determined with the cecal mucosal samples of 5 piglets at each time point (Figure 1G). In total, 6,847 cecal proteins were detected (Supplementary Table S4) with 1,453 differentially expressed proteins (Supplementary Table S5). In total, 916 out of the 1,453 differentially expressed protein genes were also found in the scRNA-seq data set (Supplementary Table S6). The 916 proteins were clustered into 3 groups (Figure 1G) and their functions were enriched by Metascape online. Group 1 included 303 proteins that were higher at d0 and d1, then decreased from d7 during cecal development (Figure 1G; Supplementary Table S7). These proteins were mainly associated with catabolic and metabolic process, correlating the functions of CCs which matched the scRNA-seq data (Figure 1G). Protein levels in group 2 gradually decreased from d1 to d21 with functions related to nuclei and organic compound metabolism, also correlating to CCs (Figure 1G). Group 3 protein levels were lower from d0 to d1 while higher from d7 to d21 and functions were related to defense and immune function, correlating the functions of Ims (Figure 1G). Overall, there was a good match between proteomic and scRNA-seq data.

### 3.2 Development of Cecal Ims Population

**FIGURE 2:**
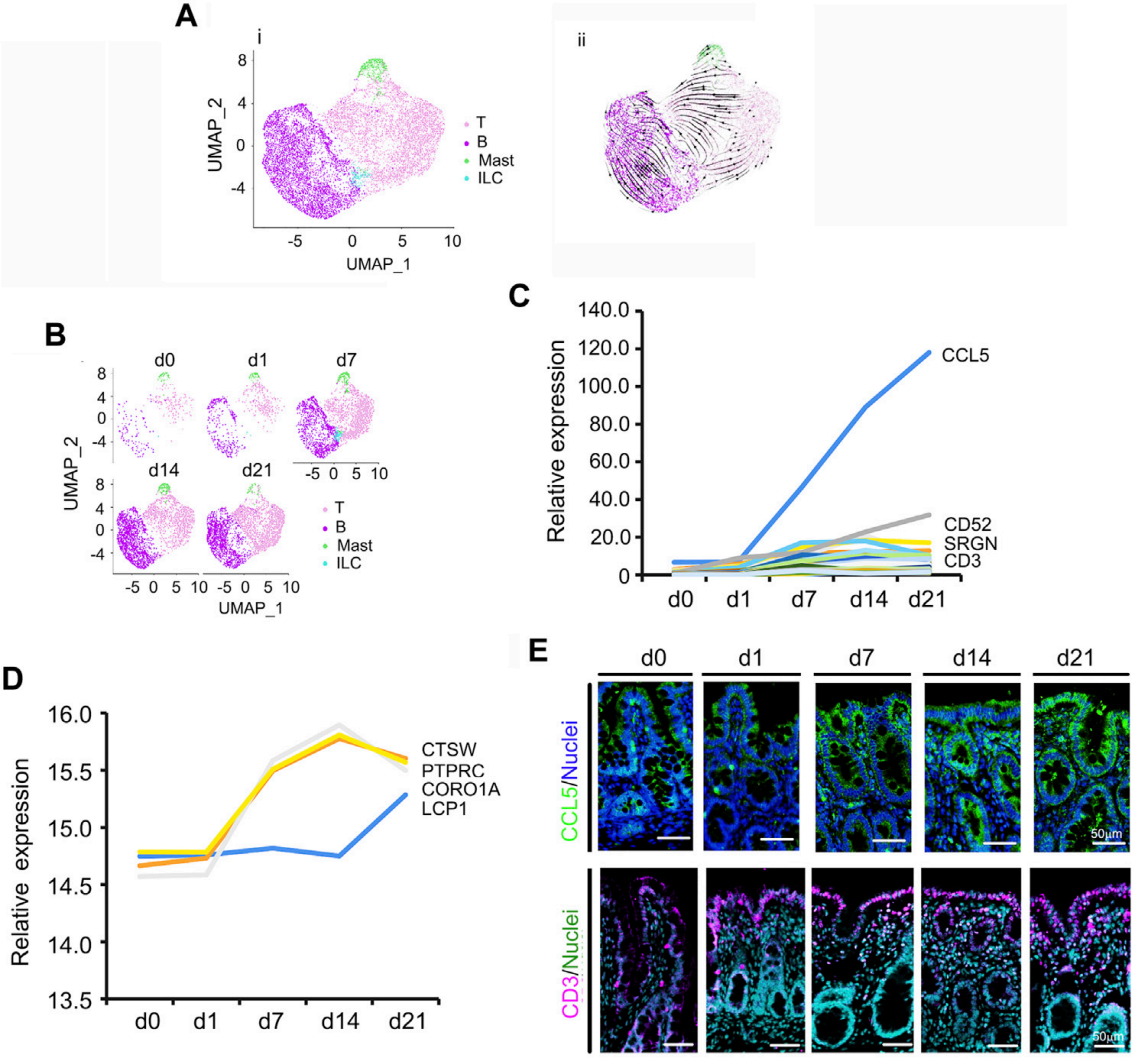
Increase in piglet cecal immune cells (Im) during the neonatal window. **(A)** Cell type clusters for Im for the cells together from the five time points by UMAP. (i) UMAP of Im single cells (points), colored by cluster assignment. (ii) RNA velocity vector projection on UMAP plot (The arrow indicated the developmental trend). **(B)** Im population changes during development (from d0 to d21). **(C)** Relative expression pattern of the top 50 specifically expressed genes in Im. The gene level was based on the expression of each gene in all the cell, and it is relative level from scRNA-seq analysis. The Y-axis presents the relative expression, and X-axis shows the time points. **(D)** Relative protein levels of some of the top 50 specifically expressed genes from the proteomic analysis. The Y-axis presents the relative expression, and X-axis shows the time points. **(E)** The protein levels of CCL5 and CD3 in the different samples at different time points according to IHF. Scale bar: 50 μm.

During neonatal development, Ims cells increased significantly (Figures 2A,B; Supplementary Table S8) from 5.12 to 61.08% during d0–d21 (Figure 1E; Figure 2B). Figure 2A showed the UMAP map for Ims for different cell types in whole while Figure 2B presented the UMAP maps for Ims at each time point with different cell types. There were four subclusters of Ims in the neonatal cecum: T cells, B cells, mast cells and innate lymphoid cells (ILC; Figures 2A,B; Supplementary Figure S2A–C). The developmental trajectory of these Ims started from ILC to T cells and B cells; however, mast cells showed a different developmental trajectory (Figure 2A; Supplementary Figure S2C; RNA velocity). T cells, and B cells continued increasing from d0 to d21; while mast cells increased from d0 to d14 then decreased; however, ILC increased from d0 to d7, then fell quickly in proportion at d14 and d21 (Figure 2B). To search the correlation of gene expression patterns and cell populations, the expression levels of the top 50 specifically expressed genes from these Ims were determined. The expression of most of these 50 genes gradually increased from d0 to d21 (Figure 2C; Supplementary Table S9), which matched the increase in proportion of the Ims. Moreover, the protein levels showed a similar trend to that of gene expression (Figure 2D; Supplementary Table S6) in the proteomics data. According to IHF analysis, protein levels of *CCL5* and *IL6* increased from d0 and d1 to d21, which confirmed the proteomics data and scRNA-seq data (Figures 2C,E; Supplementary Figure S2E,F). Figure 2C was the relative expression pattern of the top 50 specifically expressed genes in Ims. Figure 2E showed the IHF staining for *CCL5* and *CD3*. Concurrently, the proportion of *CD3* (part of T-cell receptor/CD3 complex; involved in T-cell development and signal transduction) positive cells increased from d0 to d 21, which further confirmed the proteomics data and scRNA-seq data (Figures 2C,E; Supplementary Figure S2G).

### 3.3 Unique Maturation of CCs

**FIGURE 3:**
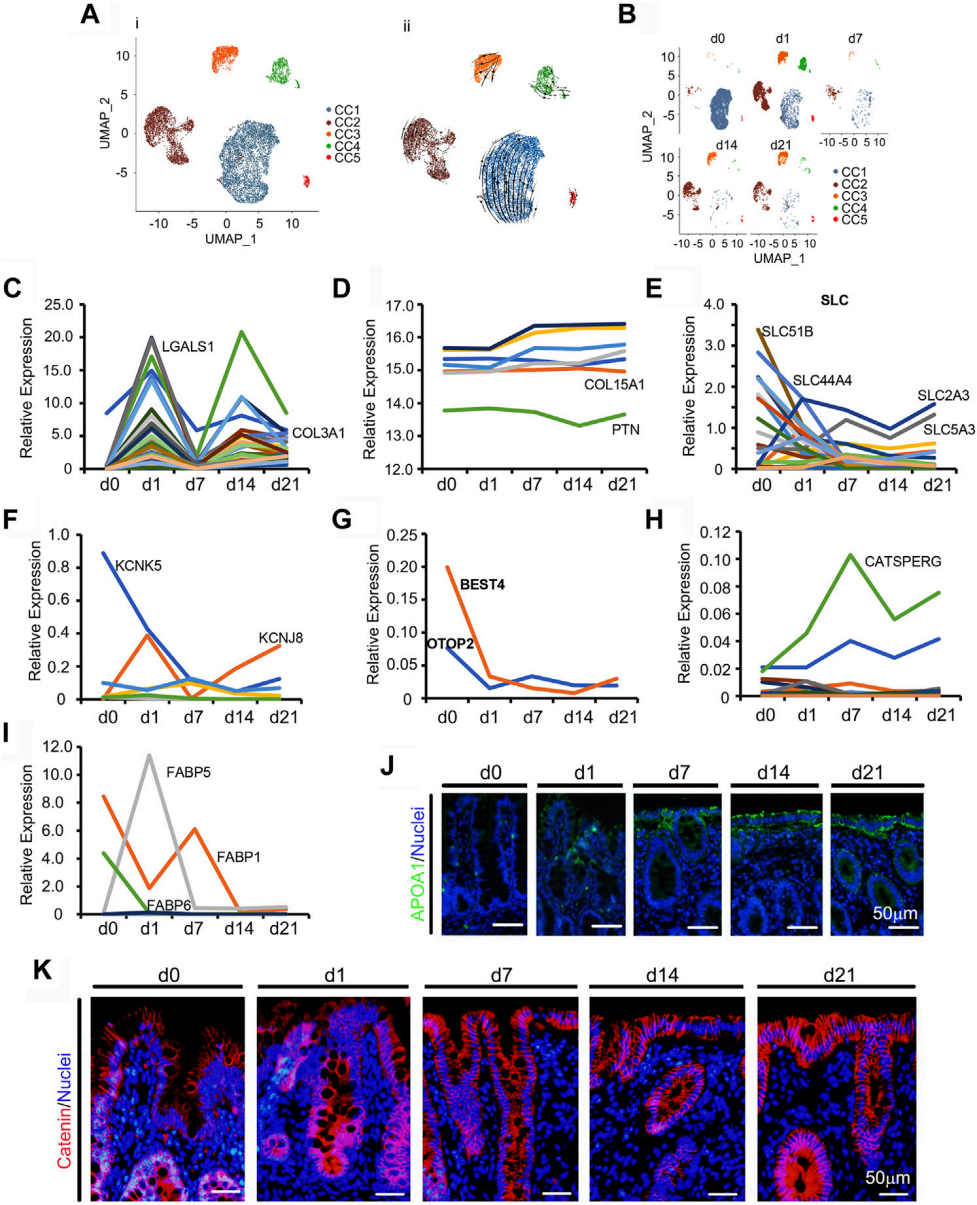
Differentiation of piglet cecal enterocytes (CC) during the neonatal window. **(A)** Cell type clusters for CC for the cells together from the five time points by UMAP: CC1 to CC5. (i) UMAP of CC single cells (points), colored by cluster assignment. (ii) RNA velocity vector projection on UMAP plots (The arrow indicated the developmental trend). **(B)** CC population changes during development (from d0 to d21). **(C)** The relative expression pattern of the top 50 specifically expressed genes in CC. The gene level was based on the expression of each gene in all the cell, and it is relative level from scRNA-seq analysis. The Y-axis presents the relative expression, and X-axis shows the time points. **(D)** The relative protein levels of some of the top 50 specifically expressed genes from the proteomic analysis. The Y-axis presents the relative expression, and X-axis shows the time points. **(E)** The relative expression pattern of solute carrier family genes. The gene level was based on the expression of each gene in all the cell, and it is relative level from scRNA-seq analysis. The Y-axis presents the relative expression, and X-axis shows the time points. **(F)** The relative expression pattern of potassium channel subfamily genes. The gene level was based on the expression of each gene in all the cell, and it is relative level from scRNA-seq analysis. The Y-axis presents the relative expression, and X-axis shows the time points. **(G)** The relative expression pattern of BEST4 and OTOP2. The gene level was based on the expression of each gene in all the cell, and it is relative level from scRNA-seq analysis. The Y-axis presents the relative expression, and X-axis shows the time points. **(H)** The relative expression pattern of cation channel family genes. The gene level was based on the expression of each gene in all the cell, and it is relative level from scRNA-seq analysis. The Y-axis presents the relative expression, and X-axis shows the time points. **(I)** The relative expression pattern of fatty acid binding protein family genes. The gene level was based on the expression of each gene in all the cell, and it is relative level from scRNA-seq analysis. The Y-axis presents the relative expression, and X-axis shows the time points. **(J)** Protein levels of APOA1 in the different samples at different time points according to IHF. Scale bar: 50 μm. **(K)** Protein levels of catenin in the different samples at different time points according to IHF. Scale bar: 50 μm.

Unlike the developmental potential of neonatal enterocytes in the ileum (Meng et al., 2021), neonatal ceca followed a unique pattern of development (Figures 3A,B; Supplementary Figures S3A–C; Supplementary Table S10). During the neonatal window, CCs grew and differentiated very quickly (Figures 1D,E; Figures 3A,B; Supplementary Figure S3A). Figure 3A showed the UMAP map for CCs for different cell types in whole while Figure 3B presented the UMAP maps for CCs at each time point with different cell types. There were 5 subclusters of CCs: CC1, CC2, CC3, CC4, and CC5 (Figures 3A,B; Supplementary Figure S3A). Overall, the proportion of CCs quickly decreased from d1 to d7, then increased at d14 and d21 (Figure 1E; Figure 3B). The majority of CCs were grouped to the CC1 subcluster (Figures 3A,B). The proportion of CC1 continued decreasing from d0 to d21, while the proportion of CC2, CC3, CC4, and CC5 increased from d0 to d1 and then decreased from d1 to d7; however, there was an increase from d7 to d14 followed by a decrease to d21, which presented a complicated development trend (Figure 3B). Gene enrichment analysis showed that the main functions of the marker genes in CC1 and CC2 were closely associated with enterocytes; while the main functions of the marker genes in CC3 and CC4 were closely associated with blood vessel formation; meanwhile, the main functions of the marker genes in CC5 were related to neuron development. Owing to the wide function of these CCs, RNA velocity and unsupervised pseudotime analyses showed similar trends (Figure 3A; Supplementary Figure S3C).

To correlate gene expression patterns and cell population changes, the expression levels of the top 50 specifically expressed genes from CCs were analyzed. There were two expression peaks at d1 and d14 during neonatal development (Figure 3C). Furthermore, the protein levels of some of the 50 genes from the proteomics analysis showed a decreasing trend or very little change (Figure 3D). The main function of the large intestine (cecum and colon) is to reabsorb water, other small molecules, and fermentation to produce organic acids and other compounds (Dabareiner and White, 1997; Peterson and Artis, 2014). Gene expressions of the solute carrier family (Figure 3E), potassium channel subfamily (Figure 3F), proton channel family (Figure 3G), cation channel family (Figure 3H), and fatty acid binding protein family showed unique trends (Figure 3I) that correlated to CCs functions. The protein levels of ferritin (*FTH1*), the major intracellular iron storage protein, increased with neonatal cecal development (Supplementary Figures S3D,E). Another enterocyte protein, intestinal *FABP*, was expressed in piglet ceca at low levels (Supplementary Figures S3D, **F)** (Peterson and Artis, 2014). Levels of the absorption protein *APOA1* increased along with cecal maturation (Figure 3J; Supplementary Figure S3G). At the same time the cell junction protein catenin was more condensed at d7–d21 than that at d0–d3, which indicated that the cecum maturated with time (Figure 3K; Supplementary Figure S3H).

### 3.4 Developmental Trends of Secretory Cells

There are 4 major types of secretory cells in the small intestine mucosal epithelium: goblet, Paneth, tuft, and EECs. It has previously been reported that there are no Paneth cells in the large intestine (Parikh et al., 2019); however, there are Paneth like cells (PLCs) in the colon (Parikh et al., 2019). Similarly, we found PLCs, goblet, and EECs (no tuft cells) in the cecum of piglets in the current study (Figures 1B–E); these cells showed a different developmental trend (Figure 1E).

**FIGURE 4:**
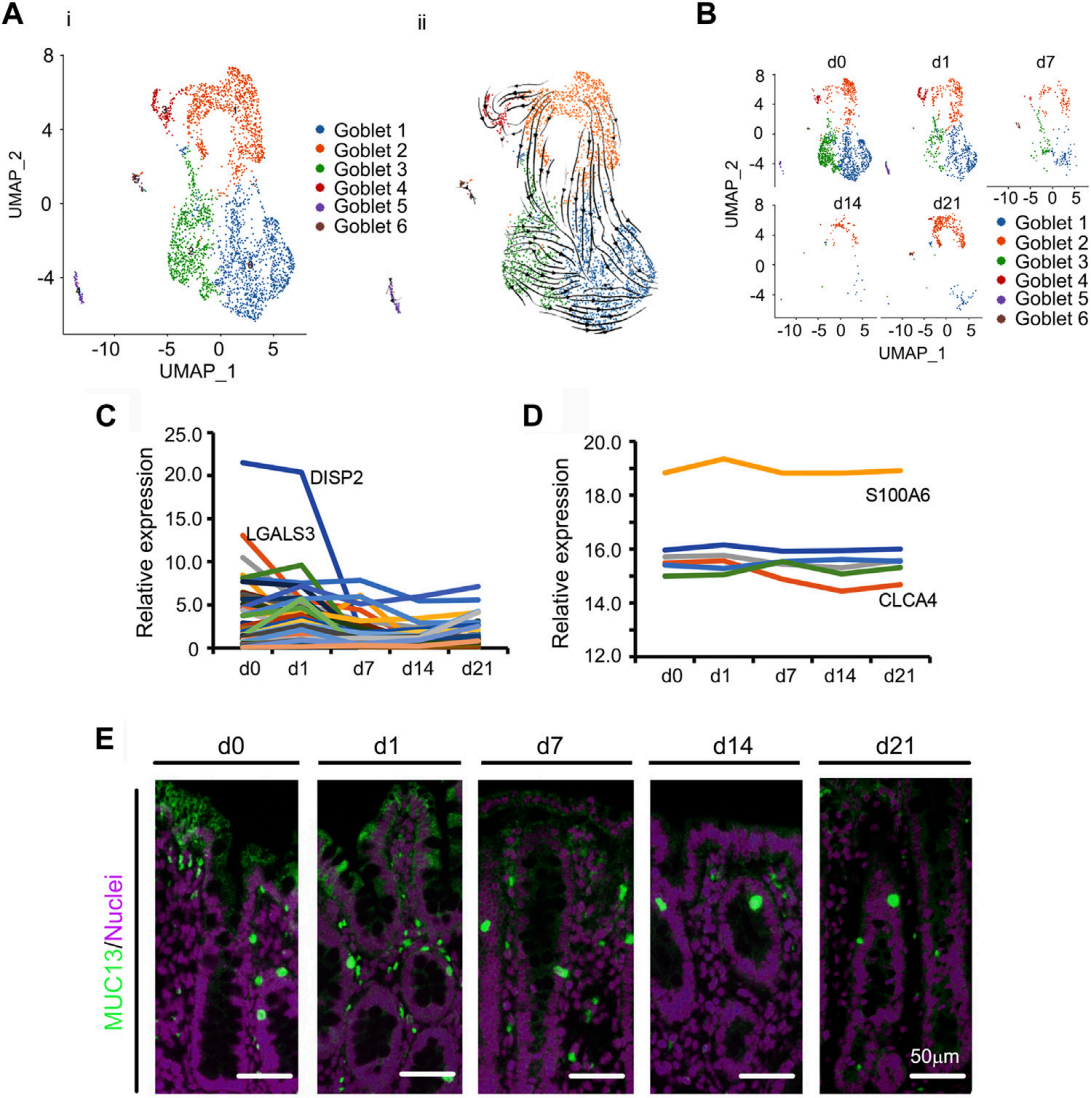
Decrease in goblet cells of piglet cecum during the neonatal window. **(A)** Cell type clusters for goblet cells for the cells together from the five time points by UMAP. (i) UMAP of goblet single cells (points), colored by cluster assignment. (ii) RNA velocity vector projection on UMAP plot (The arrow indicated the developmental trend). **(B)** Decrease in goblet cell population during development (from d0 to d21). **(C)** The relative expression pattern of the top 50 specifically expressed genes in goblet cells. The gene level was based on the expression of each gene in all the cell, and it is relative level from scRNA-seq analysis. The Y-axis presents the relative expression, and X-axis shows the time points. **(D)** The relative protein levels of some of the top 50 specifically expressed genes from the proteomics analysis. The Y-axis presents the relative expression, and X-axis shows the time points. **(E)** Protein levels of MUC13 in different samples at different time points according to IHF. Scale bar: 50 μm.

In the small intestine, goblet cells synthesize and secrete mucus (Birchenough et al., 2015), which assists with the elimination of gut content and also immune defense (Kim and Ho, 2010). In the current study, cecal goblet cells were the second most prolific at d0 (Figure 1E; Supplementary Figure S1B). Specifically, there were 6 subclusters of goblet cells (Goblet 1, Goblet 2, Goblet 3, Goblet 4, Goblet 5, and Goblet 6; Figures 4A,B; Supplementary Figure S4A–C; Supplementary Table S11). Figure 4A showed the UMAP map for goblet cells for different cell types in whole while Figure 4B presented the UMAP maps for goblet cells at each time point with different cell types. Goblet 1 had the most cells while Goblet 4, Goblet 5, and Goblet 6 contained only a small proportion of cells. Goblet 2 acted as progenitor cells as demonstrated by the RNA velocity and pseudotime analysis (Figure 4A; Supplementary Figure S4C). Although the total proportion of goblet cells continuously declined (Figure 1E; Figure 4B), those of Goblet 2 cells initially decreased and then increased at d21 (Figure 4B). The top 50 specifically expressed goblet cell genes followed the same trend as the proportion of goblet cells, decreasing during d0–d21; this was especially relevant to *COX2*, which was most highly expressed in goblet cells (Figure 4C). Furthermore, the protein levels of some of these 50 genes followed the same trend as gene expression in goblet cells (Figure 4D) in the proteomics analysis. At the same time, the goblet markers *MUC13*, and *TFF3* were also present in goblet cells (Figure 4E; Supplementary Figures S4D–F).

**FIGURE 5:**
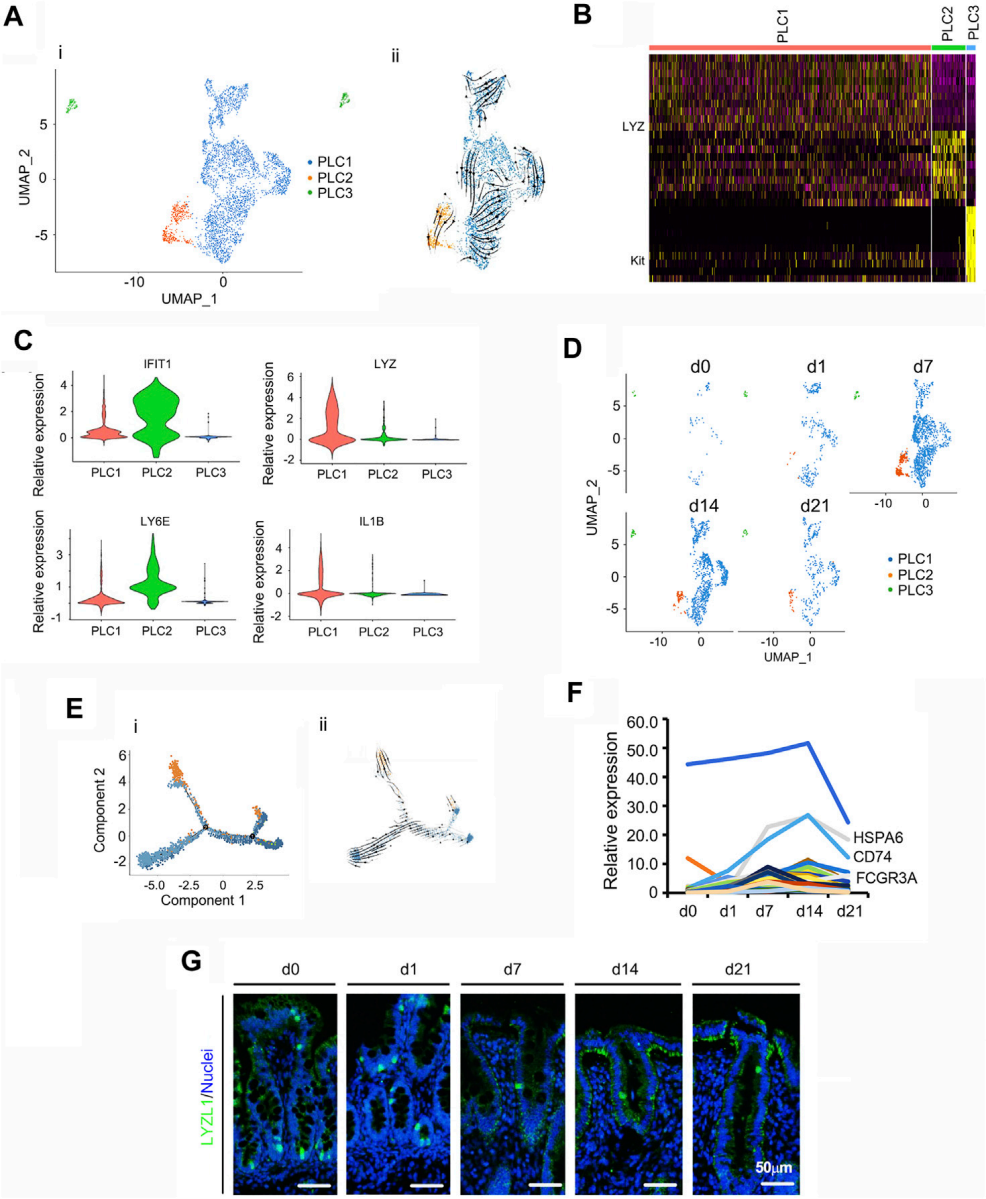
Summary of scRNA-seq data for Paneth like cells (PLCs). **(A)** Cell type clusters for PLCs for the cells together from the five time points by UMAP. (i) UMAP of PLCs single cells (points), colored by cluster assignment. (ii) RNA velocity vector projection on UMAP plot (The arrow indicated the developmental trend). **(B)** The heatmap of the top 25 marker genes in each cluster of PLCs. **(C)** Expression pattern of marker genes in different clusters of PLCs. **(D)** Decrease in PLCs population during development (from d0 to d21). **(E)** Trajectory reconstruction of PLCs based on cell clusters following pseudotime analysis. (i). Monocle plot. (ii). RNA velocity vector projection on a monocle plot (The arrow indicated the developmental trend). **(F)** The relative expression pattern of the top 50 specifically expressed genes in PLC. The gene level was based on the expression of each gene in all the cell, and it is relative level from scRNA-seq analysis. The Y-axis presents the relative expression, and X-axis shows the time points. **(G)** Protein levels of LYZL1 in the different samples at different time points according to IHF. Scale bar: 50 μm.

PLCs, are highly specialized cells with intensive secretory activity that are located at the base of crypts within the small intestine; their ability to produce copious secretions is owing to their extensive endoplasmic reticulum and Golgi structures (Bevins and Salzman, 2011; Clevers and Bevins, 2013). Also, within the small intestine, Paneth cells perform important antimicrobial functions as their large granules have the ability to release antimicrobial molecules including peptides (Bevins and Salzman, 2011; Clevers and Bevins, 2013). In the current study, there were 3 subclusters of PLCs with different marker genes (PLC1, PLC2, and PLC3; Figures 5A–E; Supplementary Table S12). Figure 5A showed the UMAP map for PLCs for different cell types in whole while Figure 5D presented the UMAP maps for PLCs at each time point with different cell types. The majority of PLC were in subcluster PLC1. Overall, the proportion of PLCs gradually increased from d0 to d7, then reduced at d4 and d21 (Figure 5D). The expression levels of the top 50 specifically expressed genes in PLCs followed cell proportion trends, especially for Reg4, HSPA6 and CD74 (Figure 5F). Protein levels of the PLCs marker *LYZ* (Sasaki et al., 2016; Haber et al., 2017) followed the trend of its gene expression pattern, which confirmed the scRNA-seq data (Figure 5G; Supplementary Figure S5A).

**FIGURE 6:**
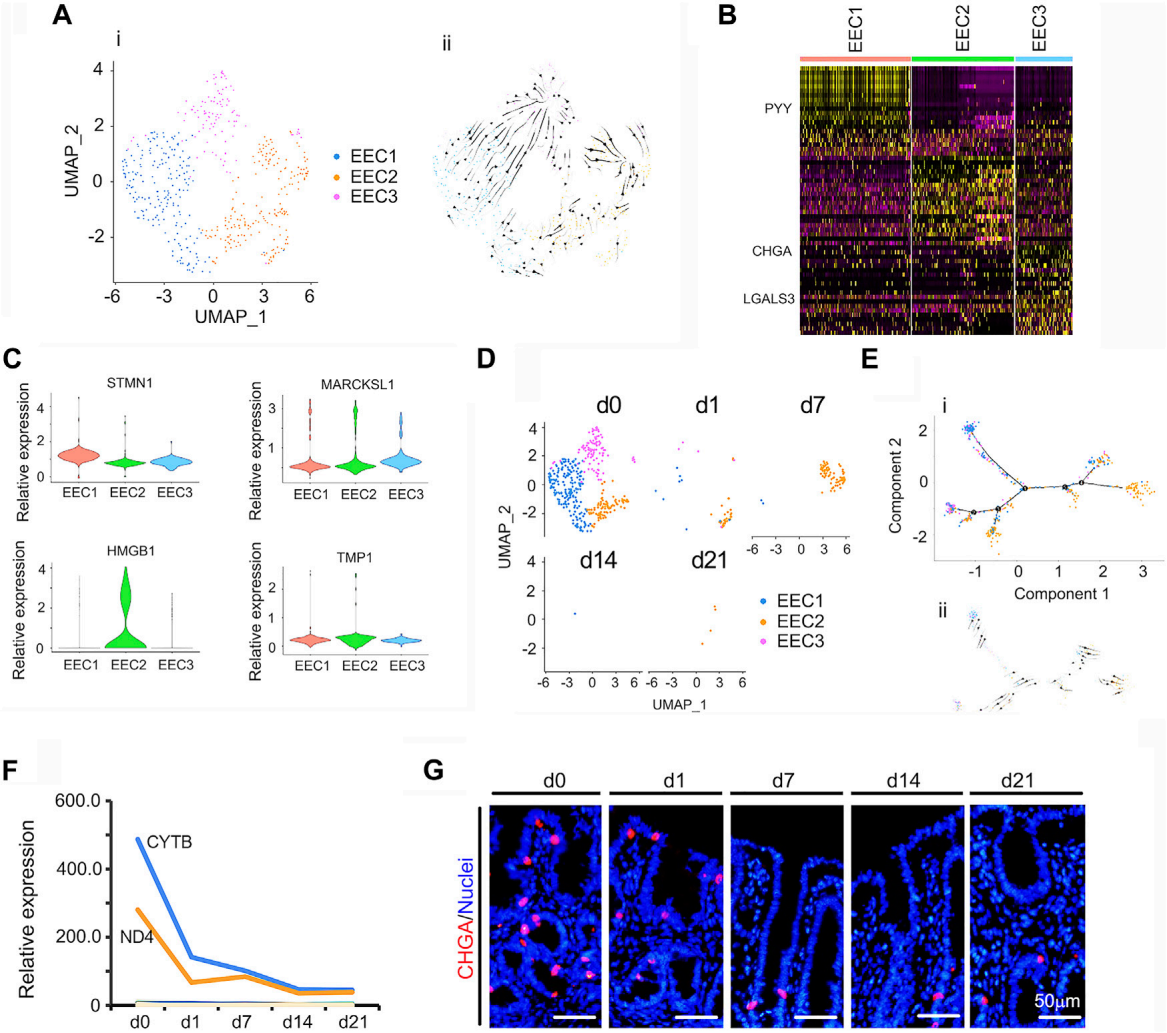
Summary of scRNA-seq data for EECs. **(A)** Cell type clusters for EECs for the cells together from the five time points by UMAP. (i) UMAP of EECs single cells (points), colored by cluster assignment. (ii) RNA velocity vector projection on UMAP plots (The arrow indicated the developmental trend). **(B)** Heatmap of the top 25 marker genes in each cluster of EECs. **(C)** Expression pattern of marker genes in different EECs clusters. **(D)** EECs population decrease during development (from d0 to d21). **(E)** EECs trajectory reconstruction based on cell clusters following pseudotime analysis. (i) Monocle plot. (ii) RNA velocity vector projection on a monocle plot (The arrow indicated the developmental trend). **(F)** The relative expression pattern of the top 50 specifically expressed genes in EECs. The gene level was based on the expression of each gene in all the cell, and it is relative level from scRNA-seq analysis. The Y-axis presents the relative expression, and X-axis shows the time points. **(G)** Protein levels of CHGA in different samples at different time points according to IHF. Scale bar: 50 μm.

Intestinal EECs are known to be key sensory cells (Furness et al., 2013; Gribble and Reimann, 2016) that secrete various hormones and play important roles in nutrient and microbial metabolism (Haber et al., 2017; Worthington et al., 2018). In the small intestine, EECs are members of many overlapping sub-clusters (Habib et al., 2013; Gribble and Reimann, 2016). Cells expressing Sct, Cck, Gcg, or GIP are commonly, and respectively, called S, I, L, and K cells (Haber et al., 2017). In the current investigation, EEC in the neonatal piglet cecal epithelium were classed into 3 subclusters (Figures 6A–E; Supplementary Table S13). Figure 6A showed the UMAP map for EECs for different cell types in whole while Figure 6D presented the UMAP maps for EECs at each time point with different cell types. The proportion of EECs continued to fall from d0 to d21 (Figure 6D) and the expression levels of the top 50 specifically expressed genes in EEC followed the same trend (Figure 6F). Protein levels of *CHGA* (the marker gene of EECs) followed its gene expression pattern, which confirmed the scRNA-seq data (Figure 6G; Supplementary Figure S5B).

### 3.5 Regulation of Cecal Cell Maturation

**FIGURE 7:**
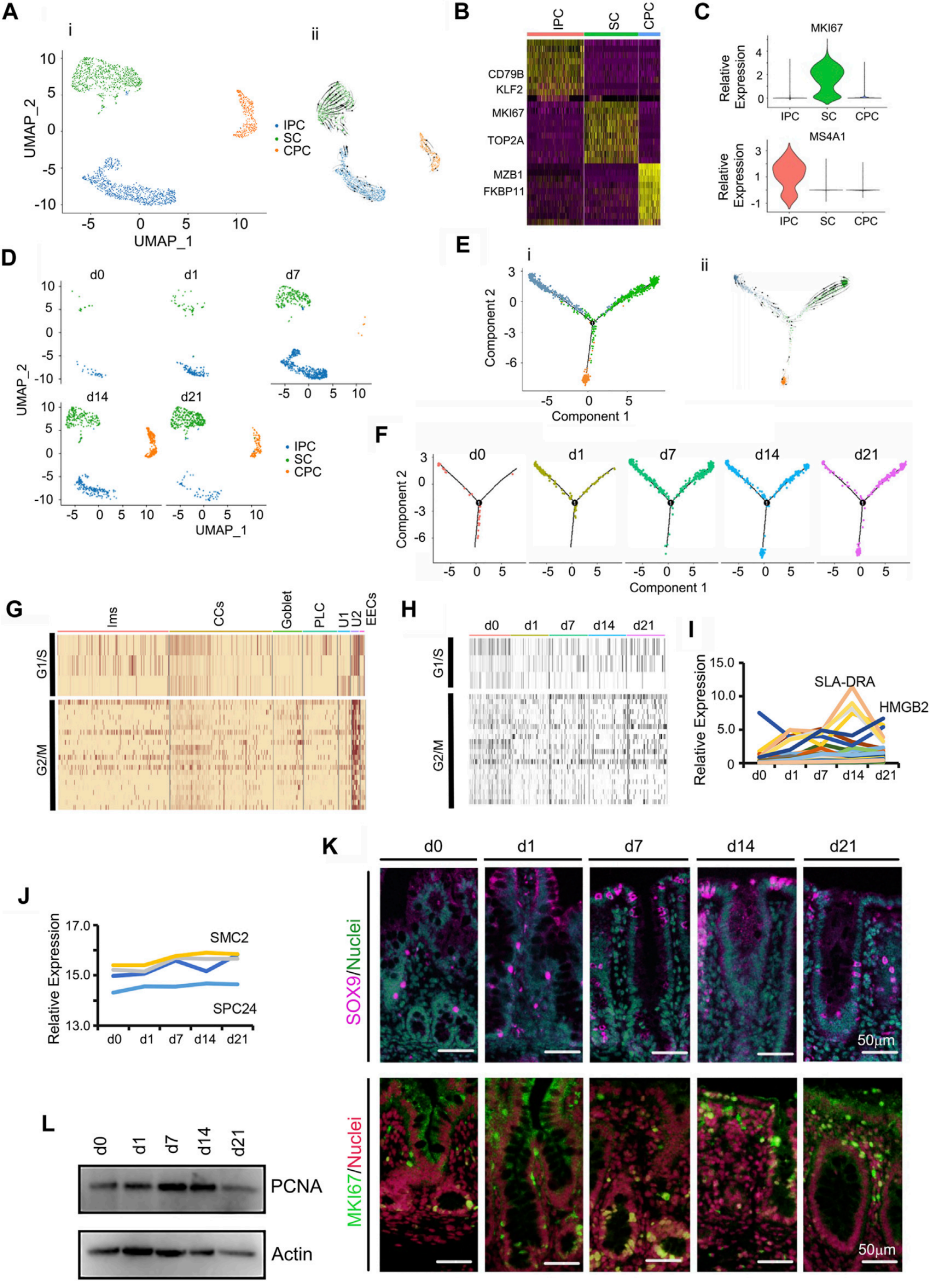
Increase in piglet cecum undifferentiated cells (U1/U2) during the neonatal window. **(A)** Cell type clusters for U1/U2 for the cells together from the five time points by UMAP. (i) UMAP of undifferentiated single cells (points), colored by cluster assignment. (ii) RNA velocity vector projection on UMAP plots. **(B)** Heatmap of the top 25 marker genes in each cluster of U1/U2. **(C)** Expression pattern of marker genes in different clusters of U1/U2. **(D)** U1/U2 population changes during development (from d0 to d21). **(E)** Trajectory reconstruction of U1/U2 based on cell clusters according to pseudotime analysis. (i). Monocle plot. (ii). RNA velocity vector projection on a monocle plot (The arrow indicated the developmental trend). **(F)** Monocle images for different samples at 5 time points. **(G)** Heatmap of cluster genes for the cell cycle in different clusters of cells. **(H)** Heatmap of cluster genes for the cell cycle in samples at different time points. **(I)** The relative expression pattern of the top 50 specifically expressed genes in U1/U2. The gene level was based on the expression of each gene in all the cell, and it is relative level from scRNA-seq analysis. The Y-axis presents the relative expression, and X-axis shows the time points. **(J)** The relative protein levels of some of the top 50 specifically expressed genes from the proteomic analysis. **(K)** Protein levels of SOX9 and Ki67 in different samples at different time points according to IHF. Scale bar: 50 μm. **(L)** Protein levels of PCNA in different samples at different time points according to WB.

Intestinal cell renewal is orchestrated by intestinal stem cells (SCs) through their production of highly proliferative progenitor cells (Figures 7A–F; Supplementary Table S14); these cells form an undifferentiated cell pool with the potential to develop into all types of mature cecal cells: CCs, PLCs, goblet, Ims, and EECs (Williams et al., 2001; Labib et al., 2004; Peterson and Artis, 2014). Figure 7A showed the UMAP map for undifferentiated cells (U1/U2) for different cell types in whole while Figure 7D presented the UMAP maps for U1/U2 at each time point with different cell types. During neonatal development, these cecal undifferentiated cells underwent specific increases from 0.69 to 11.79% during d0–d21 (Figure 1E; Figure 7D). There were 3 subclusters of undifferentiated cells: stem cells (SCs) expressed stem cell markers; immune progenitor cells (IPCs) expressed proliferation markers and immune markers; and cecum enterocyte progenitor cells (CPCs) expressed proliferation markers and enterocyte markers (Figure 7B).

Expression of genes involved in cell cycle regulation was higher in undifferentiated cells in comparison with CCs or secretory cells (Figure 7G), and was unchanged from d0 to d21 (Figure 7H). To determine correlation of gene expression pattern and cell population, the expression levels of the top 50 differentially expressed genes from these undifferentiated cells were determined. The expression of most of these 50 genes gradually increased from d0 to d14 (Figure 7I), which matched the increase in proportion of the undifferentiated cells. Moreover, the protein levels from proteomics data showed a similar trend to that of gene expression (Figure 7J). The proportion of stem cell marker *SOX9* (Verdile et al., 2019) increased at d7-d21 (Figure 7K; Supplementary Figure S5C). Similarly, the proportion of cell proliferation marker *Ki67* positive cells increased at d7-d21 (Figure 7K; Supplementary Figure S5D). Concurrently, *PCNA* protein levels increased from d1 to d14 using WB analysis, which confirmed the proteomics data and scRNA-seq data (Figure 7L; Supplementary Figure S5E).

**FIGURE 8:**
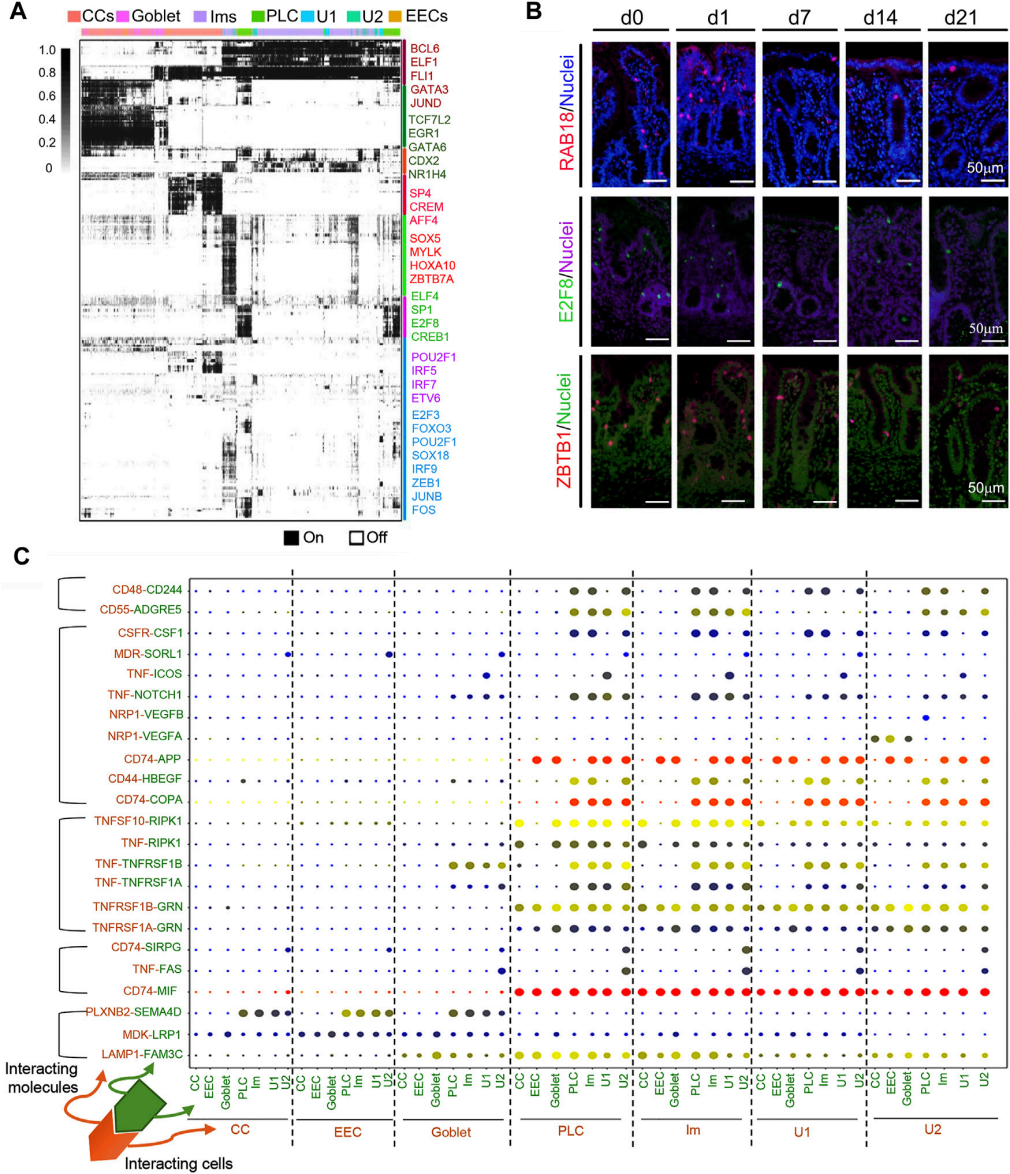
Multiple regulatory networks in cecal cells in the neonatal window. **(A)** SCENIC binary regulon activity heatmap depicting different clusters of cecal epithelium cell enriched regulons. Columns contain single cells while the rows indicate regulons. “On” = active; “Off” = inactive. **(B)** IHF images of TFs between d0 and d21. Scale bar: 50 μm. **(C)** Overview of selected ligand-receptor interactions; *p*-values are indicated by circle size; the scale is on the right (for permutation test, see Methods). Means of average expression levels of interacting molecule 1 (cluster 1) and interacting molecule 2 (cluster 2) are indicated by color. Assays were performed at the mRNA level, but here they are extrapolated to protein interactions.

Gene regulatory network [GRNs; transcriptional factors (TFs)] (Aibar et al., 2017) analysis of the various types of cecal epithelia exposed several master regulators within each cell population (Figure 8A; Supplementary Table S15). Notably, the binary regulon activity heatmap indicated that CC, and Im had a predominantly high expression of regulons while the secretory cell clusters had relatively low regulon expression (Figure 8A). There was also some overlapping of regulon activity for different cluster of cells. TF protein levels confirmed their gene expression (Figure 8B; Supplementary Figure S5F–H; Supplementary Figure S6), including *ZBTB1*, *RAB18*, *E2F8*, *Pou2AF1*, and *FOX O 3a*.

Cell-cell communication takes place through ligand-receptor complexes; such coordination is important for multiple biological activities, including development, and differentiation (Vento-Tormo et al., 2018; Efremova et al., 2020). CellPhoneDB analysis (www.CellPhoneDB.org), based on ligand-receptor interacting pairs, was used in the exploration of cellular interaction at the cecal cell interface (Figure 8C; Supplementary Table S16). Overall, ligand-receptor interaction was higher in U1/U2, Im, and PLC, both with each other and with other cells (CCs, goblet, and EECs) compared with the interaction of CCs, goblet, and EECs with each other, or with U1/U2, Im, and PLCs (Figure 8C). The more significant pairs were CD74-APP, CD74-COPA, and CD74-MIF, followed by TNFSF10-RIPK1, TNFSF1B-GNR, and LAMP1-FAM3C. Some of these pairs have been reported to have broad functions; for example, CD74-MIF is involved in several biological processes associated with the modulation of inflammation, cardiac function, and tumor formation (Soppert et al., 2018).

GPCRs, TGF-β signaling, and BMP signaling greatly affect intestinal development (Haber et al., 2017; Gao et al., 2018). In total, 35 GPCRs were expressed in the cecal epithelium (Supplementary Figures S7A, B). Some of these receptors such as signal sequence receptor subunit 4 (SSR4) and CCR7 were specifically expressed in some cell types or at some time points (Supplementary Figures S7A,B). The TGF-β signaling pathway members TGFBR2, TGFBR1, SMAD7, SMAD4, SMAD2, and BMP signaling pathway members BMP2K and BMP4 were also specifically expressed in some types of cecal cells or at some time points (Supplementary Figures S7C, D). Our results suggest that, during the neonatal window, these factors may affect cecal cell development.

### 3.6 Microbiota Involved in Neonatal Cecal Cell Maturation

**FIGURE 9:**
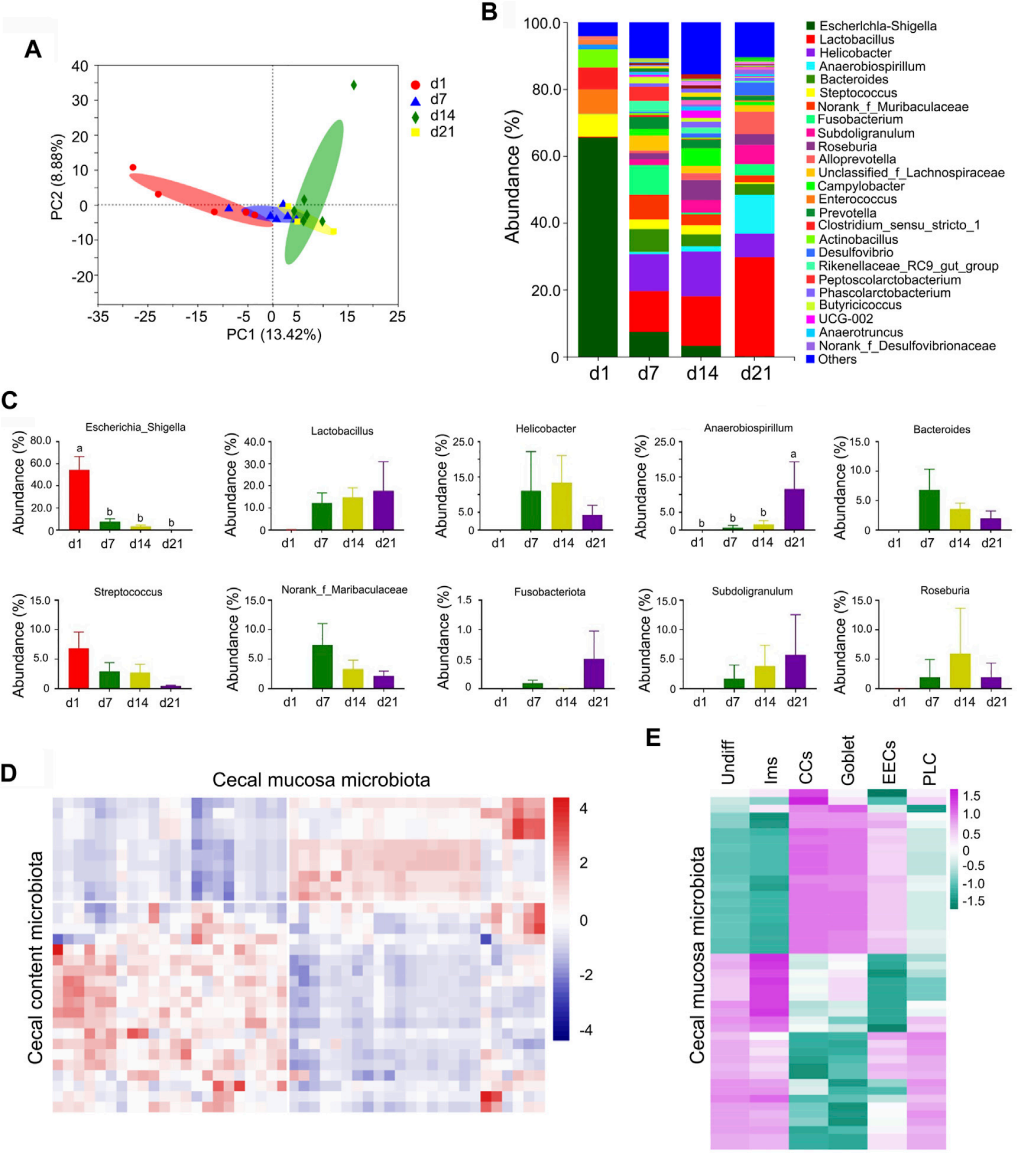
Changes in cecal mucosa microbiota. **(A)** The PCA of the microflora in cecal mucosal samples from different time points. The X-axis shows the PC1 while the Y-axis presents the PC2. **(B)** Differences of bacterial abundance at the genus level. The X-axis shows the time points while the Y-axis presents the relative proportion. **(C)** Relative proportion of the 10 major microbiotas. The X-axis shows the time points while the Y-axis presents the relative proportion. **(D)** Pearson correlation of the proportion of different microbiota in cecal mucosa and cecal content. **(E)** Pearson correlation of the proportion of different microbiota in cecal mucosa and the proportion of different clusters of cells.

At the point of birth, a neonate moves from a sterile uterine environment to an external microbe-rich environment (Palmer et al., 2007; Costello et al., 2009; Dominguez-Bello et al., 2010; Koenig et al., 2011; Renz et al., 2011). Shortly after birth, almost no microbiota was found in piglet cecal mucosa or content, while with development, the diversity of cecal microbiota (both mucosa and content) increased to initiate their influence on intestinal cell development (Figures 9A–C; Supplementary Figures S8A–E) (Palmer et al., 2007; Costello et al., 2009; Dominguez-Bello et al., 2010; Koenig et al., 2011; Renz et al., 2011). Figure 9A showed the PCA analysis of the microflora in cecal mucosal samples from different time points. Figure 9B present the differences of bacterial abundance at the genus level. The relative proportion of the 10 major cecal mucosal microbes (at the genus level) changed during the neonatal window (Figure 9C; Supplementary Table S17), as did the relative proportion of the 10 major cecal content microbes (Supplementary Figure S8E; Supplementary Table S18). In both cecal content and mucosa, one of the major microbe populations was *lactobacillus* (Figure 9C; Supplementary Figure S8E). Moreover, there was a good correlation between cecal content microbes and mucosal microbes (Figure 9D; Supplementary Table S19). The cecal mucosa microbes and the cecal cell population (sRNA-seq) were well correlated (Figure 9E; Supplementary Table S20). The cecal content microbes and the cecal cell population (sRNA-seq) were also well correlated (Supplementary Figure S8F; Supplementary Table S21).

The “beneficial” microbiota *lactobacillus* (Zhang et al., 2020) was first observed in swine cecal content at d1 and from there increased in proportion to become the major microbiota at d21. Meanwhile the proportion of other major microbiota either decreased or fluctuated over the same period (Supplementary Figure S8E). *Lactobacillus* started to appear in the swine cecal mucosa at d7 and increased to become the major microbiota at d21, while other major microbiota either decreased or fluctuated over the same period of development (Figure 9C). This may be because the experimental piglets were raised solely on maternal milk, without interventions such as antibiotics, additives, or immunization.

**FIGURE 10:**
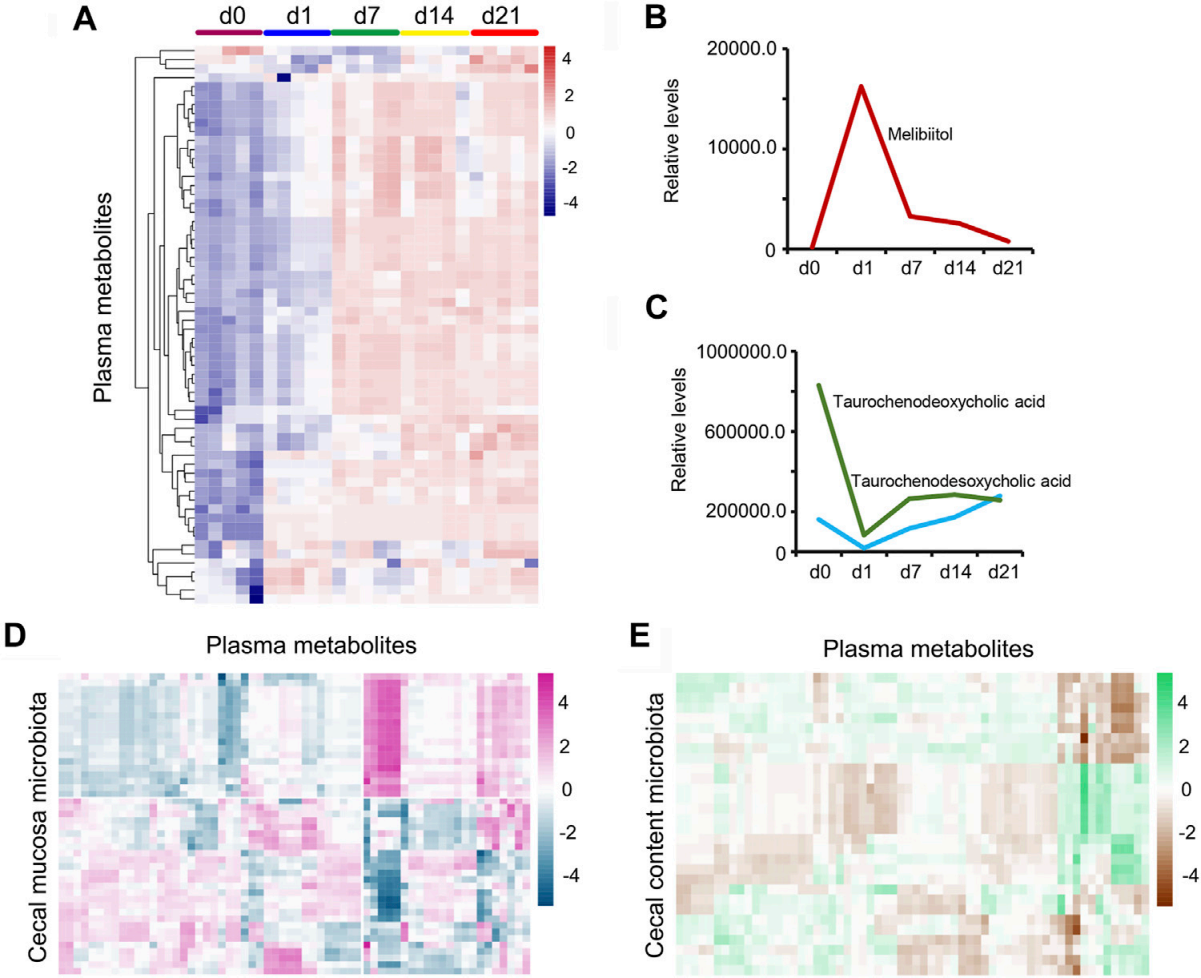
Piglet plasma metabolism. **(A)** Heatmap for the continuing increase in the expression levels of 62 metabolites from d0 to d21. **(B)** Plasma concentration of Melibiitol at different time points. The X-axis shows the time points while the Y-axis presents the relative levels. **(C)** Plasma concentration of taurochenodeoxycholic acid and taurochenodesoxycholic acid at different time points. The X-axis shows the time points while the Y-axis presents the relative levels. **(D)** Pearson correlation of the proportion of cecal mucosa microbiota and the concentration of plasma metabolites. **(E)** Pearson correlation of the proportion of cecal content microbiota and the concentration of plasma metabolites.

Furthermore, piglet plasma metabolism was determined by LC/MS, and 5,388 metabolites were found in the plasma samples (Supplementary Table S22). Sixty-two metabolites continued increasing from d1 to d21 (Figure 10A; Supplementary Table S23). Melibiitol, belonging to galactose metabolism, quickly increased from d0 to d1, then decreased to d21 (Figure 10B). The two bile acids metabolites taurochenodeoxycholic acid and taurochenodesoxycholic acid decreased from d0 to d1, then increased to d21, which indicated the developmental potential of the intestine (Figure 10C). The plasma metabolites and cecal mucosa microbiota showed a more profound correlation (Figure 10D; Supplementary Table S24) than that of plasma metabolites and cecal content microbiota (Figure 10E; Supplementary Table S25). The data indicated that cecal cells and microbiota were involved in the changes taking place during plasma metabolism.

## 4 Discussion

To our knowledge, this is the first presentation of a large-scale scRNA-seq study of the piglet cecal cells during the neonatal developmental period. The study has revealed novel cellular diversity and subtype-specific gene expression in different types of cecal cells.

It is well known that the cecum is a critical place for absorption of water and electrolyte, and is lacking villi and a brush border with little or no intrinsic digestive function (Dabareiner and White 1997; Elmentaite et al., 2021). In current study, we found that just after born (d0-d7) the cecum had similar brush bord as the small intestine with villi expression in piglets. However, when the piglets grow up, the brush became flat with less villi expression (d14-d21). In current study, the single cell RNA-seq analysis showed that the different types of cells changed during this developmental window to represent the cecum growth.

Using scRNA-seq analysis, we have characterized 6 major types of cecal cells: U1/U2, Ims, CCs (Parikh et al., 2019), goblet, PLCs, and EECs with specific marker genes. Moreover, these types of cells had specific developmental potentials. CCs matured with a gradual decrease in proportion; however, Ims developed with a continuing increase in proportion. Meanwhile, goblet cells reduced in proportion from d0 to d14; PLCs increased in proportion from d0 to d7 then decreased at d14 and d21; and EECs decreased in proportion during the neonatal developmental period. The proteomics data matched the scRNA-seq with almost half of the changed proteins being highly expressed at d0 and d1 and exhibiting a decrease from d7 to d21, correlating to the developmental trend of CCs; however, approximately 50% of the proteins were expressed at low levels at d0 and d1 while they increased from d7 to d21 and were correlated with Im developmental potential. The decrease in the proportion of CCs indicated that the brush became flat in cecum as the piglets growing up. And the increase in the proportion of Ims suggested that immune function of cecum became stronger during the piglet growth (Mowat and Agace, 2014). Goblet cells play important roles in the mucus secretion and protection from gut content (Kim and Ho, 2010; Birchenough et al., 2015), in current study, the proportion of goblet cells decreased from d0 to d14 in the swine cecum. The Paneth cells were not found in the piglet cecum, however, Paneth like cells (PLCs) have been detected in the piglet cecum as early report (Parikh et al., 2019), that had secretory activity to perform important antimicrobial functions (Bevins and Salzman, 2011; Clevers and Bevins, 2013). In current study, the proportion of PLCs continued to increase from d0 to d7, then decreased at d14 and d21 in piglet cecum. EECs, the sensory cells, play important roles in hormone secretion and nutrient and microbial metabolism (Haber et al., 2017; Worthington et al., 2018). In current study, EECs were found to be a small portion of cells in piglet cecum and continued to decrease in the proportion from d0 to d21. Recently, it has been found that although EECs are a small group of cells which play crucial role in intestinal function, and they are regulated by many molecular regulators (Gehart et al., 2019).

Cell-type-specific TFs, GPCRs, and members of TGF-β and BMP signaling pathways are known to have vital roles in intestinal cell development, both during the fetal stage and in response to pathogens (Haber et al., 2017; Gao et al., 2018). During the neonatal period, the current study showed that cecal cell type differentiation was regulated by cell-intrinsic changes to regulatory programs: ligand-receptor pairs, and the above listed factors. Ligand-receptor complexes are intimately involved in cell-cell communication, a crucial event during a wide range of biological processes including development, differentiation, and inflammation (Aibar et al., 2017; Efremova et al., 2020). We found a few important ligand-receptor pairs such as CD74-APP, CD74-COPA, and CD74-MIF, followed by TNFSF10-RIPK1, TNFSF1B-GNR, and LAMP1-FAM3C that have a broad range of biological functions (Maharshak et al., 2010; Soppert et al., 2018).

The large intestine is the main reservoir for the trillions of commensal bacteria that inhabit the intestine and play critical roles in fermentation to produce short chain fatty acids and other molecules which are essential for health (Mowat and Agace, 2014). Moreover, microbiota plays vital role in the shaping of intestinal development (Williams et al., 2001; Peterson and Artis, 2014). As the piglets matured, the microbial diversity of the cecal content and mucosa increased dramatically. It was very interesting to note that beneficial microbiota, such as *lactobacillus*, was the major group in both cecal content and mucosa. This may be due to the consumption of maternal milk by the piglets. Maternal milk is rich in bioactive substances, immunoglobulins, and relatively large protein particles that are critical for intestinal, and even whole organism development (Pasternak et al., 2016; Chen et al., 2018; Skrzypek et al., 2018). Furthermore, the cecal mucosal microbiota and content microbiota were positively correlated, and also showed strong correlation with different types of cecal cells and plasma metabolites. Very importantly, the cecal mucosal microbiota showed strong correlation with plasma metabolites. All the data indicated that microbiota may help the cecum development in piglets.

In summary, we found that cecum development in piglet with different type of cells maturation and changes in the proportion. And many regulators play important roles in the cecum cell development, and cecal microbiota is involved in the regulation of cecal development. Our study for the first time increases knowledge of cecal development under normal conditions at the single cell level. These data may increase our understanding of cecal development under normal or pathological conditions in human health. Furthermore, the findings may be useful for developing novel interventions to optimize cecal drug delivery and metabolism.

## Data Availability

The datasets presented in this study can be found in online repositories. The names of the repository/repositories and accession number(s) can be found below: NCBI [accession: GSE163272, PRJNA688810]; Integrated Proteome Resources (accession: IPX0002622002).

## Supplementary Material

Supplementary Figure S1 Summary of single cell RNA-seq (scRNA-seq) data. **(A)** Study design to show the time points and the main determinants. **(B)** Histopathological images of swine cecum at different time points. **(C)** Quantitative data for vil1 IHF staining in Figure 1A. Y-axis shows the fold change to d0 (relative intensity, the level in d0 set at 1), X-axis presents the time points. a,b indicates significant at P<0.05. **(D)** Summary of single cell RNA seq for cecal mucosa tissue at different time points. **(E)** Heatmap of the top 25 marker genes in each cluster of cells. **(F)** The expression pattern of marker genes in different clusters of cells. **(G)** The number of cells in each cluster at each time point from d0 to d21.

Supplementary Figure S2 Additional data for immune cells (Im). **(A)** Heatmap of the top 25 marker genes in each cluster of Im. **(B)** Expression pattern of marker genes in different clusters of Im. **(C)** Trajectory reconstruction of Im based on cell clusters according to pseudotime analysis. (i). Monocle plot. (ii). RNA velocity vector projection on a monocle plot (The arrow indicated the developmental trend). **(D)** Protein levels of IL6 in different samples at different time points according to IHF. Scale bar: 50 μm. (E) Quantitative data for CCL5 IHF staining in Figure 2E. Y-axis shows the fold change to d0 (relative intensity, the level in d0 set at 1), X-axis presents the time points. a,b,c indicates significant at P<0.05. **(F)** Quantitative data for IL6 IHF staining in Supplementary Figure 2D. Y-axis shows the fold change to d0 (relative intensity, the level in d0 set at 1), X-axis presents the time points. a,b indicates significant at P<0.05. **(G)** Quantitative data for CD3 IHF staining in Figure 2E. Y-axis shows the fold change to d0 (relative intensity, the level in d0 set at 1), X-axis presents the time points. a,b indicates significant at P<0.05.

Supplementary Figure S3 Summary of scRNA-seq data for cecal enterocytes (CC). **(A)** Heatmap of the top 25 marker genes in each cluster of CC. **(B)** The expression pattern of marker genes in different clusters of CC. **(C)** Trajectory reconstruction of CC based on cell clusters following pseudotime analysis. (i). Monocle plot. (ii). RNA velocity vector projection on monocle plots (The arrow indicated the developmental trend). **(D)** The protein levels of FTH1 and FABP in different samples at different time points according to IHF. Scale bar: 50 μm. **(E)** Quantitative data for FTH1 IHF staining in Supplementary Figure 3D. Y-axis shows the fold change to d0 (relative intensity, the level in d0 set at 1), X-axis presents the time points. a,b indicates significant at P<0.05. **(F)** Quantitative data for FABP1 IHF staining in Supplementary Figure 3D. Y-axis shows the fold change to d0 (relative proportion of cells, the level in d0 set at 1), X-axis presents the time points. a,b indicates significant at P<0.05. **(G)** Quantitative data for APOA1 IHF staining in Figure 3J. Y-axis shows the fold change to d0 (relative intensity, the level in d0 set at 1), X-axis presents the time points. a,b indicates significant at P<0.05. **(H)** Quantitative data for catenin IHF staining in Figure 3K. Y-axis shows the fold change to d0 (relative intensity, the level in d0 set at 1), X-axis presents the time points. a,b indicates significant at P<0.05.

Supplementary Figure S4 Summary of scRNA-seq data for goblet cells. **(A)** The heatmap of the top 25 marker genes in each cluster of goblet cells. **(B)** The expression pattern of marker genes in different clusters of goblet cells. **(C)** Trajectory reconstruction of goblet cells based on cell clusters following pseudotime analysis. (i). Monocle plot. (ii). RNA velocity vector projection on a monocle plot (The arrow indicated the developmental trend). **(D)** IHF for the protein expression of goblet cell marker gene TFF3. **(E)** Quantitative data for MUC13 IHF staining in Figure 4E. Y-axis shows the fold change to d0 (relative proportion of cells, the level in d0 set at 1), X-axis presents the time points. a,b indicates significant at P<0.05. **(F)** Quantitative data for TFF3 IHF staining in Supplementary Figure 4D. Y-axis shows the fold change to d0 (relative proportion of cells, the level in d0 set at 1), X-axis presents the time points. a,b indicates significant at P<0.05.

Supplementary Figure S5 Quantitative data for IHF staining of LYZ1, CHGA, SOX9, Ki67, RAB18, E2F8, ZBTB1 and WB data for PCNA. **(A)** Quantitative data for LYZ1 IHF staining in Figure 5G. Y-axis shows the fold change to d0 (relative proportion of cells, the level in d0 set at 1), X-axis presents the time points. a,b indicates significant at P<0.05. **(B)** Quantitative data for CHGA IHF staining in Figure 6G. Y-axis shows the fold change to d0 (relative proportion of cells, the level in d0 set at 1), X-axis presents the time points. a,b indicates significant at P<0.05. **(C)** Quantitative data for SOX9 IHF staining in Figure 7K. Y-axis shows the fold change to d0 ((relative proportion of cells, the level in d0 set at 1), X-axis presents the time points. a,b indicates significant at P<0.05. **(D)** Quantitative data for Ki67 IHF staining in Figure 7K. Y-axis shows the fold change to d0 (relative proportion of cells, the level in d0 set at 1), X-axis presents the time points. a,b indicates significant at P<0.05. **(E)** Quantitative data for PCNA WB in Figure 7L. Y-axis shows the fold change to d0 (relative intensity, the level in d0 set at 1), X-axis presents the time points. a,b indicates significant at P<0.05. **(F)** Quantitative data for RAB18 IHF staining in Figure 8B. Y-axis shows the fold change to d0 (relative proportion of cells, the level in d0 set at 1), X-axis presents the time points. a,b indicates significant at P<0.05. **(G)** Quantitative data for E2F8 IHF staining in Figure 8B. Y-axis shows the fold change to d0 (relative proportion of cells, the level in d0 set at 1), X-axis presents the time points. a,b indicates significant at P<0.05. **(H)** Quantitative data for ZBTB1 IHF staining in Figure 8B. Y-axis shows the fold change to d0 (relative proportion of cells, the level in d0 set at 1), X-axis presents the time points. a,b indicates significant at P<0.05.

Supplementary Figure S6 Additional data for U1/U2. **(A)** IHF images of some of the TFs: Pou2AF1, and FOXO3a at different time points from d0 to d21. **(B)** Quantitative data for POU2AF1 IHF staining in Supplementary Figure 6A. Y-axis shows the fold change to d0 (relative proportion of cells, the level in d0 set at 1), X-axis presents the time points. a,b indicates significant at P<0.05. **(C)** Quantitative data for FOXO3a IHF staining in Supplementary Figure 6A. Y-axis shows the fold change to d0 (relative proportion of cells, the level in d0 set at 1), X-axis presents the time points. a,b indicates significant at P<0.05. **(D)** WB images of some of the TFs: MYBL2, and GATA6 at different time points from d0 to d21. **(E)** Quantitative data for MYBL2 WB in Supplementary Figure 6D. Y-axis shows the fold change to d0 (relative intensity, the level in d0 set at 1), X-axis presents the time points. a,b indicates significant at P<0.05. **(F)** Quantitative data for GATA6 WB in Supplementary Figure 6D. Y-axis shows the fold change to d0 (relative intensity, the level in d0 set at 1), X-axis presents the time points. a,b indicates significant at P<0.05.

Supplementary Figure S7 Expression patterns of GPCRs, members of TGF-β, and BMP signaling pathways. **(A)** Expression pattern of GPCRs in different clusters of cecal epithelium. **(B)** The expression pattern of GPCRs in samples from different time points. **(C)** Expression pattern of members of TGF-β and BMP pathways in different clusters of the cecal epithelium. **(D)** Expression pattern of members of TGF-β and BMP pathways in samples from different time points.

Supplementary Figure S8 Changes in cecal content microbiota. **(A)** Alpha index of the cecal mucosa microbiota: Shannon, Simpson, Chao1, and ACE index. **(B)** Alpha index of the cecal content microbiota: Shannon, Simpson, Chao1, and ACE index. **(C)** The PCA of the microflora in cecal content samples from different time points. The X-axis shows the PC1 while the Y-axis presents the PC2. **(D)** Differences in bacterial abundance at the genus level in cecal content. The X-axis shows the time points while the Y-axis presents the relative proportion. **(E)** Relative proportion of the 10 major microbiota in cecal content. The X-axis shows the time points while the Y-axis presents the relative proportion. **(F)** Pearson correlation of the proportion of cecal content microbiota and the proportion of different clusters of cells.

Supplementary Table S1 Primary antibodies.

Supplementary Table S2 scRNA-seq gene expression.

Supplementary Table S3 scRNA-seq marker genes for each cluster.

Supplementary Table S4 Protein expression in proteomics analysis (all).

Supplementary Table S5 Differentially expressed proteins.

Supplementary Table S6 Protein cluster in Figure 1G.

Supplementary Table S7 Protein enrichment for Figure 1G.

Supplementary Table S8 Marker genes for Im.

Supplementary Table S9 Top 50 specifically expressed genes in each cluster.

Supplementary Table S10 Marker genes for CC.

Supplementary Table S11 Marker genes for Goblet.

Supplementary Table S12 Marker genes for PLC.

Supplementary Table S13 Marker genes for EEC.

Supplementary Table S14 Marker genes for undifferentiated expressed genes.

Supplementary Table S15 Expression of TFs.

Supplementary Table S16 Ligand-receptor.

Supplementary Table S17 Relative abundance of microbiota for cecal mucosa.

Supplementary Table S18 Relative abundance of microbiota for cecal content.

Supplementary Table S19 Correlation of microbiota of cecal mucosa and cecal content.

Supplementary Table S20 Correlation of microbiota of cecal mucosa and cell population.

Supplementary Table S21 Correlation of microbiota of cecal content and cell population.

Supplementary Table S22 Plasma metabolites.

Supplementary Table S23 Continuing increased plasma metabolites during the neonatal period.

Supplementary Table S24 Correlation of microbiota of cecal mucosa and plasma metabolites.

Supplementary Table S25 Correlation of microbiota of cecal content and plasma metabolites.
