# Supplementary figures and images for: Multi-Omics Uncover Neonatal Cecal Cell Development Potentials
Source: Front Cell Dev Biol. 2022 Jul 15;10:840298. doi: 10.3389/fcell.2022.840298 (PMC9334561; doi:10.3389/fcell.2022.840298)

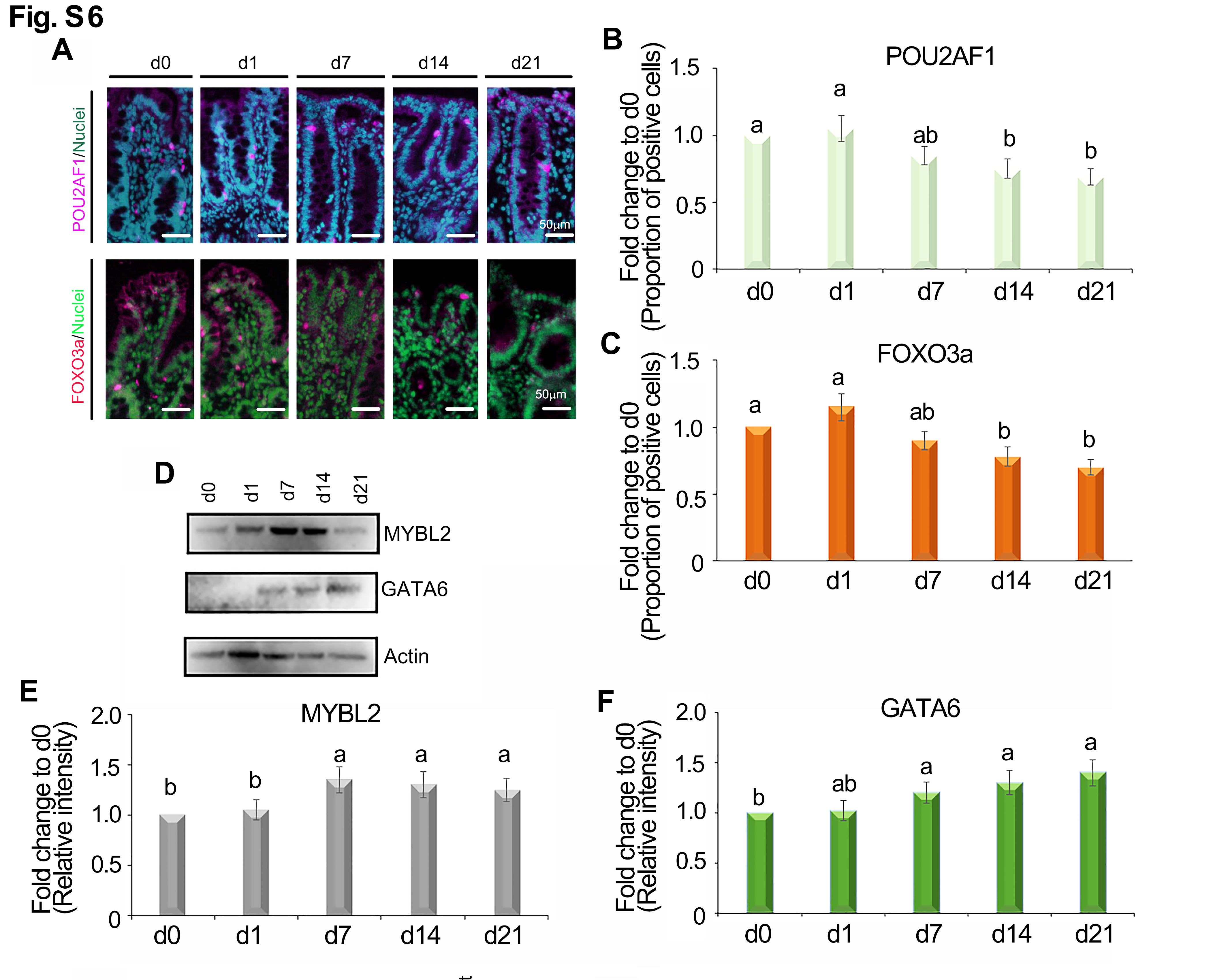

Supplement: Supplementary file 1 [file Image6.TIF]

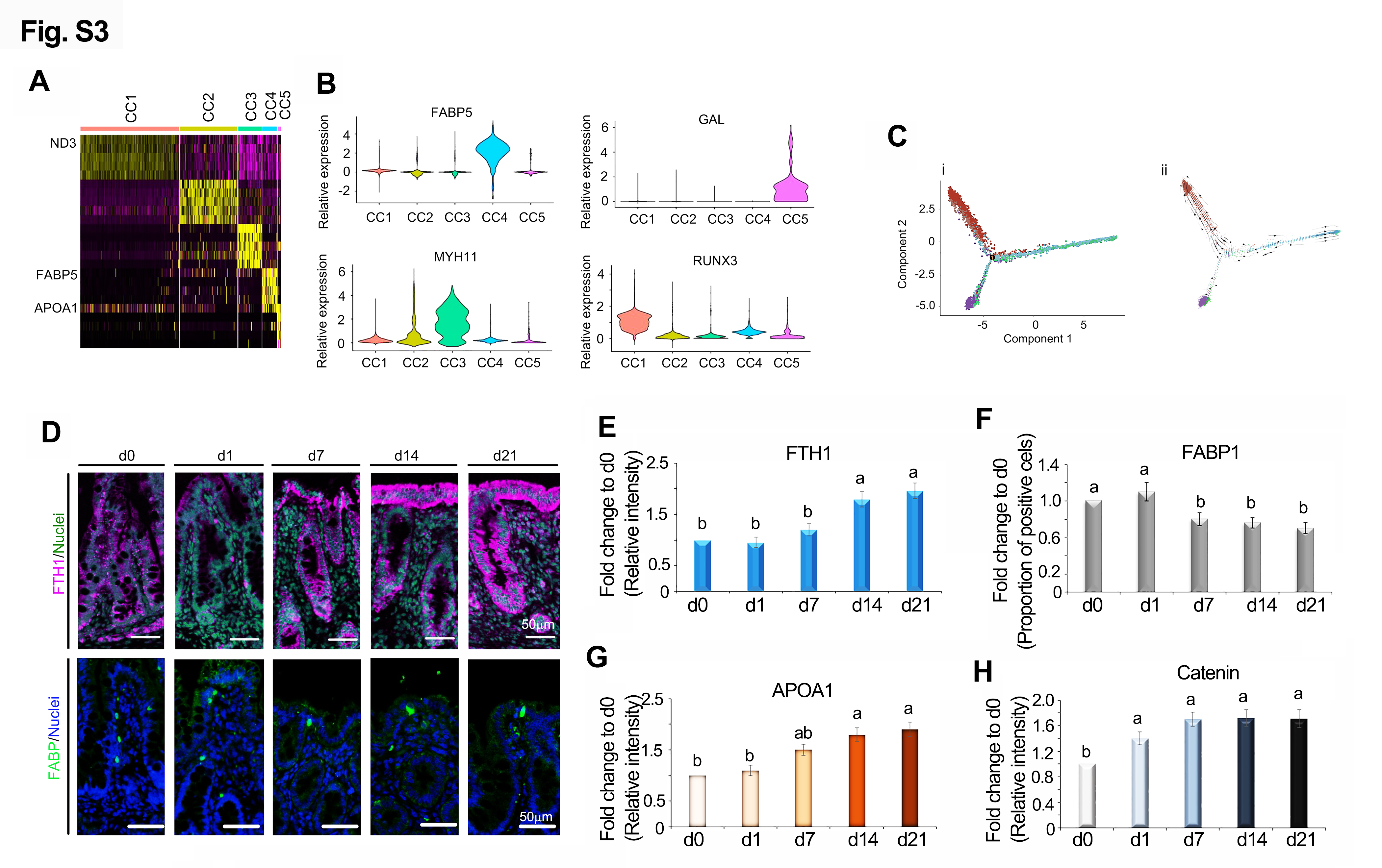

Supplement: Supplementary file 2 [file Image3.TIF]

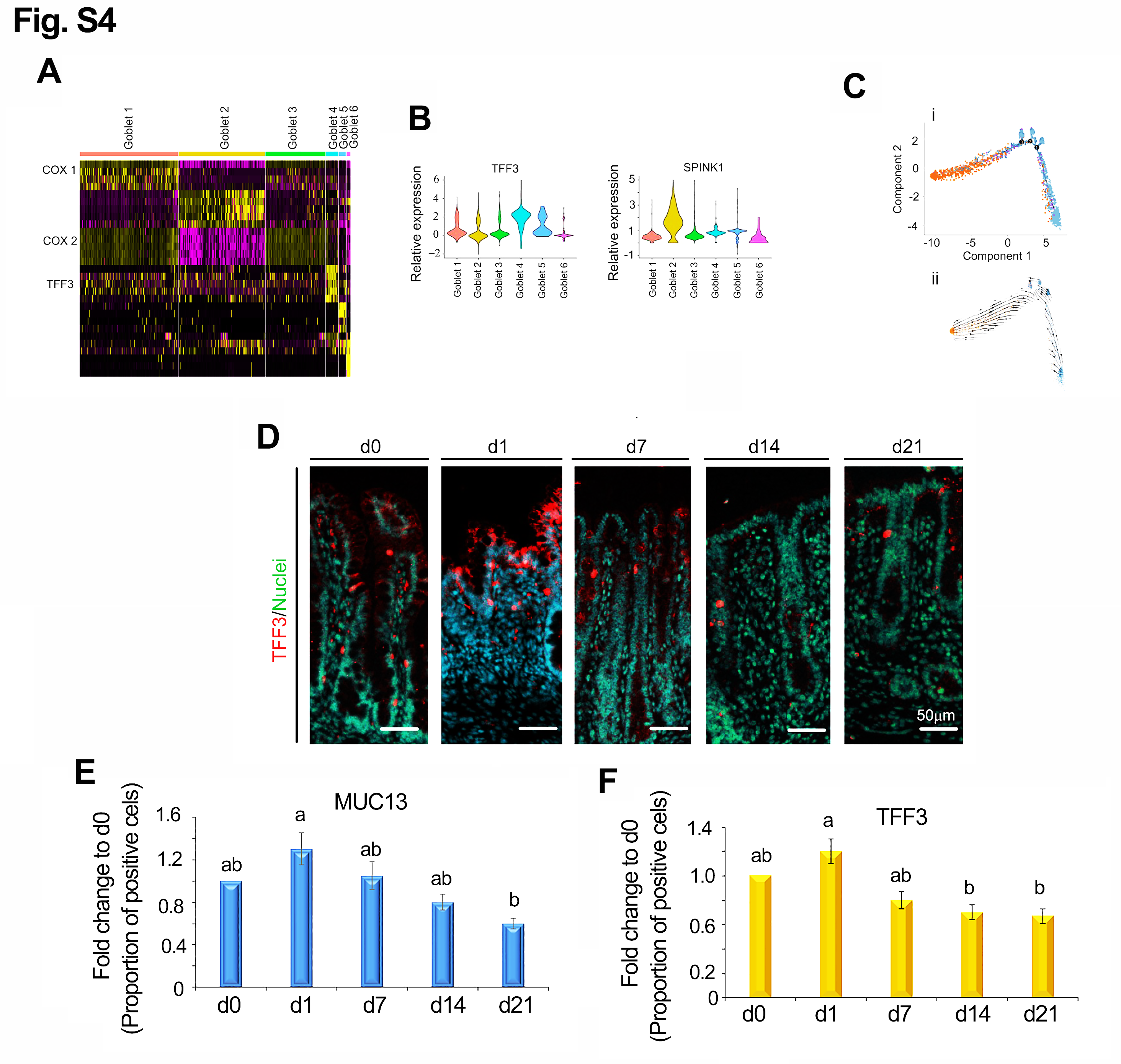

Supplement: Supplementary file 3 [file Image4.TIF]

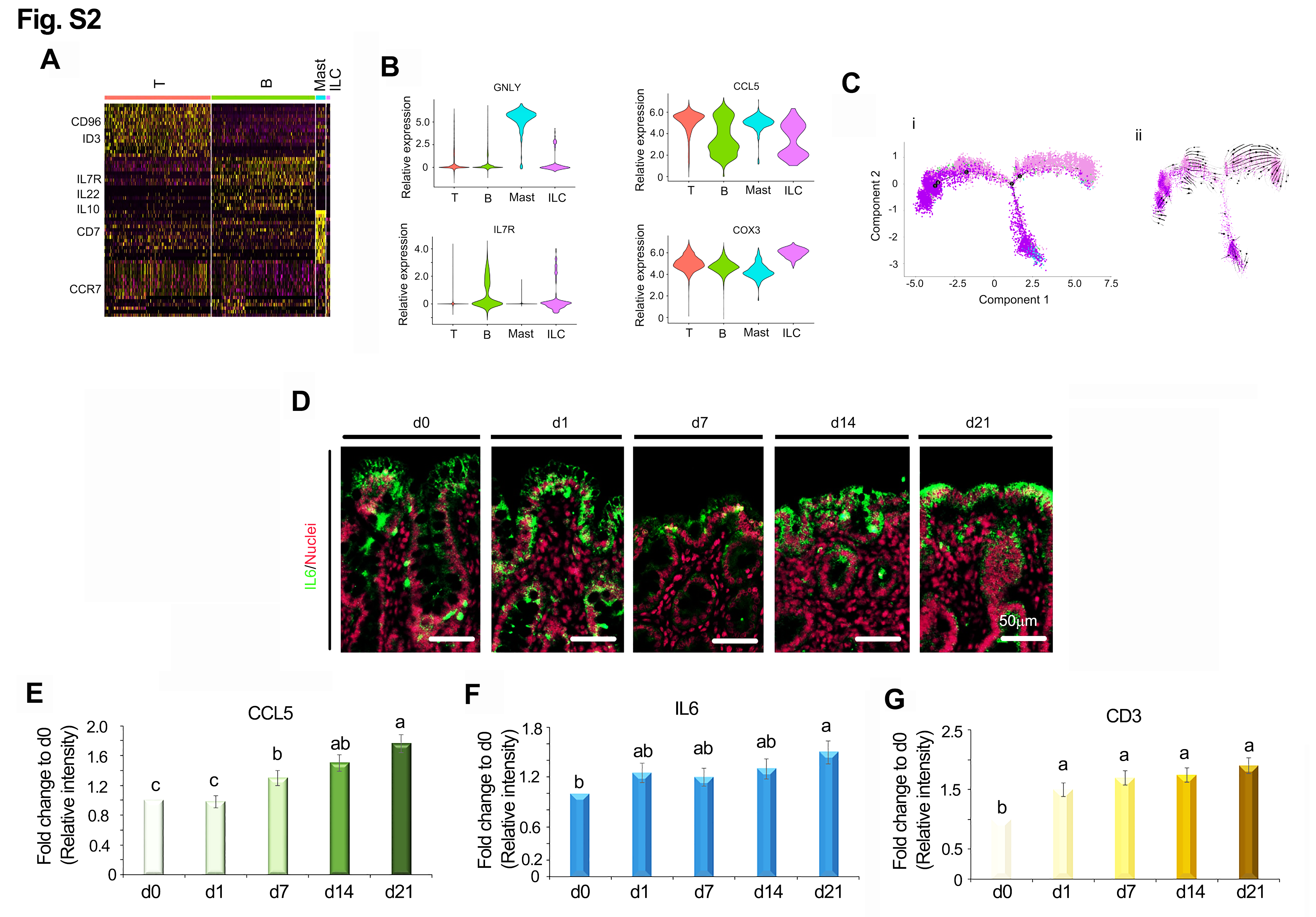

Supplement: Supplementary file 5 [file Image2.TIF]

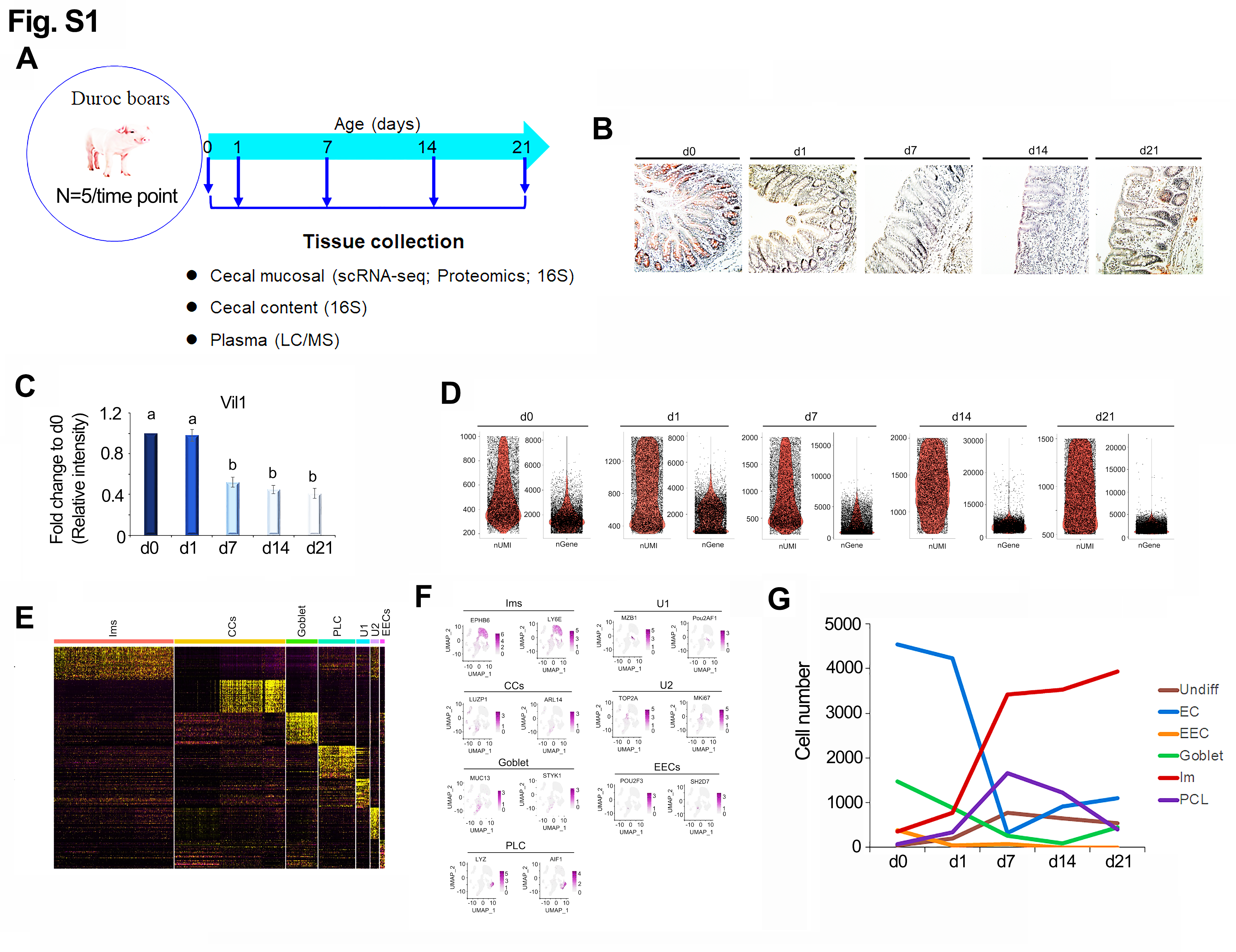

Supplement: Supplementary file 6 [file Image1.TIF]

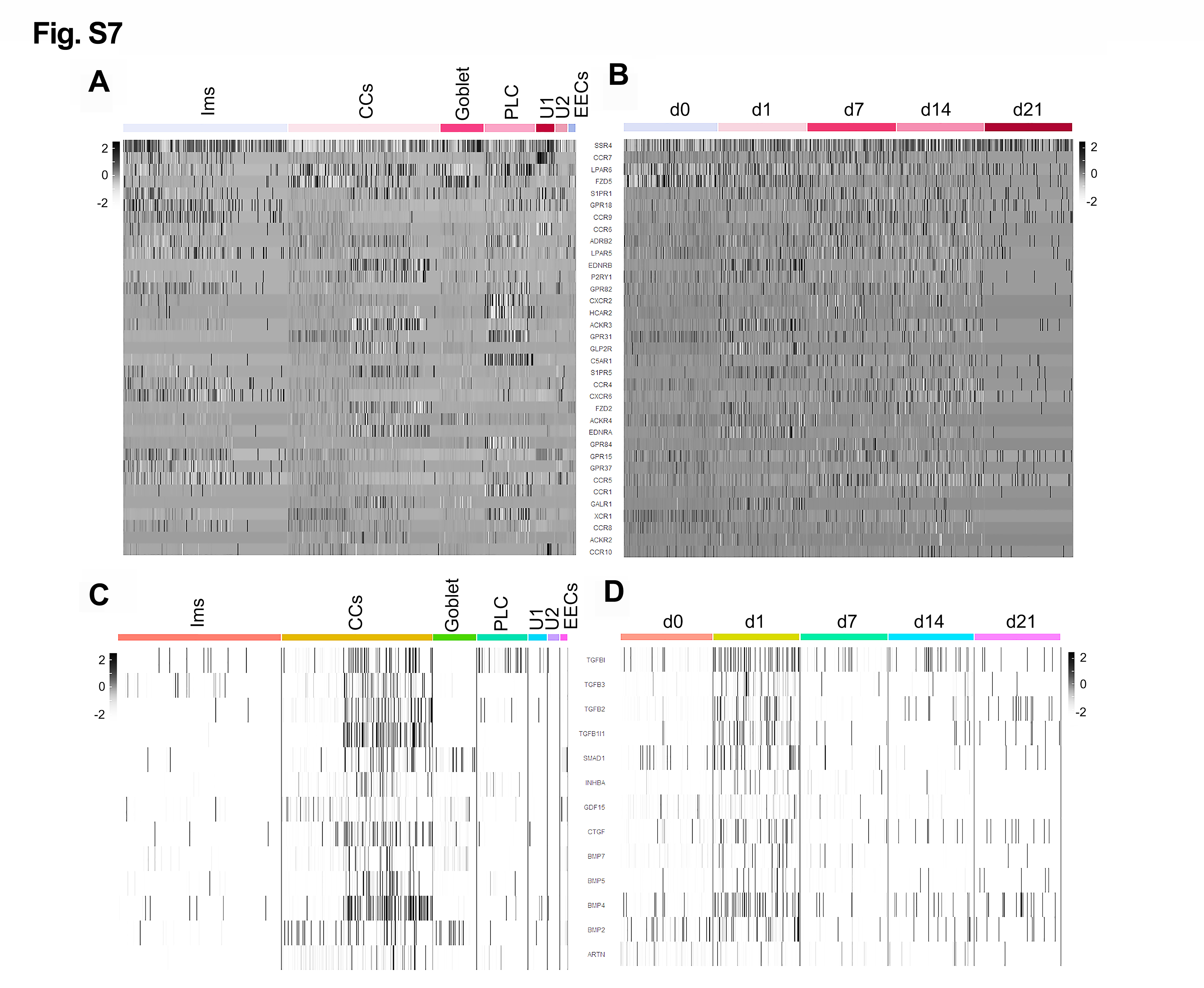

Supplement: Supplementary file 7 [file Image7.TIF]

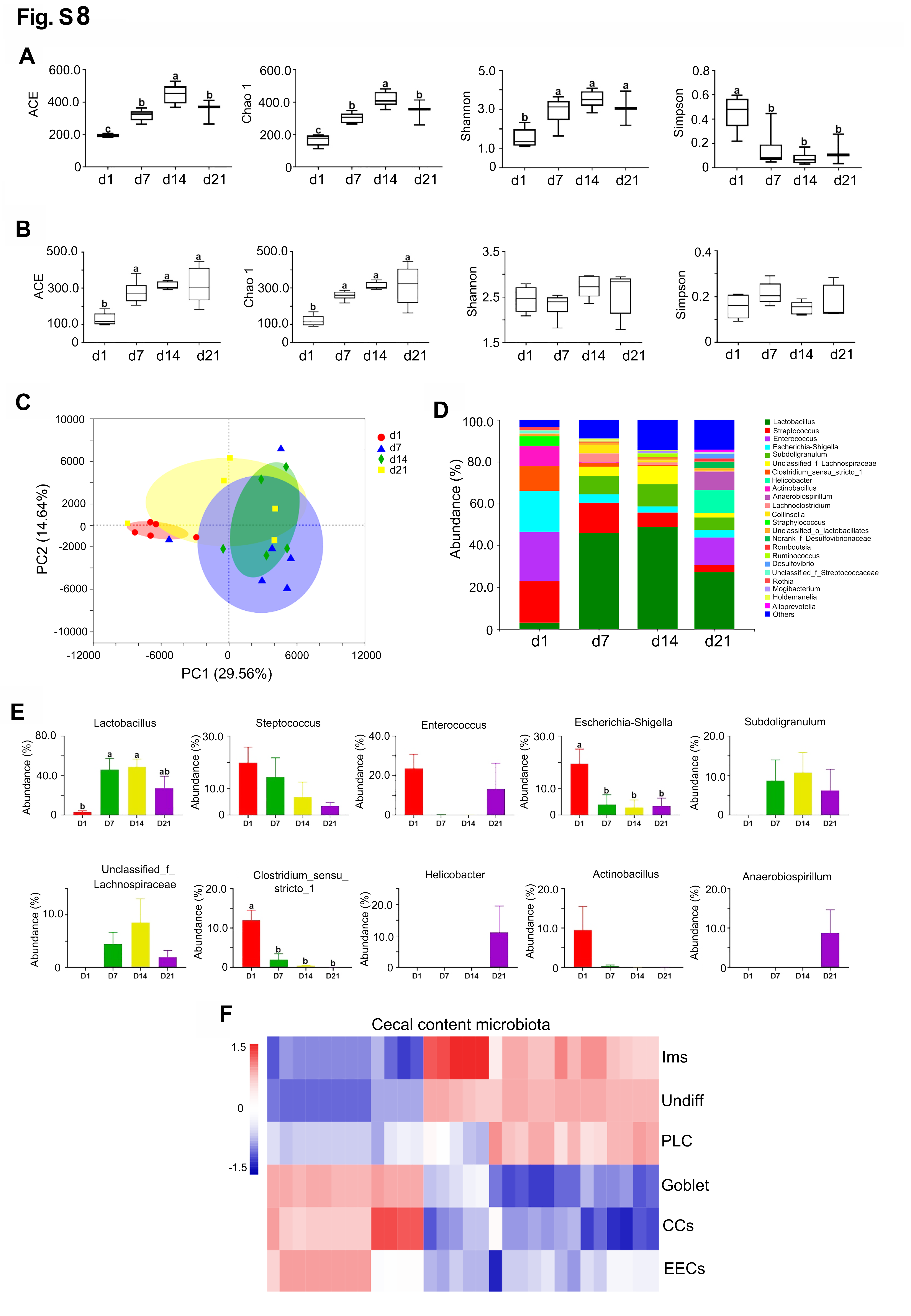

Supplement: Supplementary file 8 [file Image8.TIF]

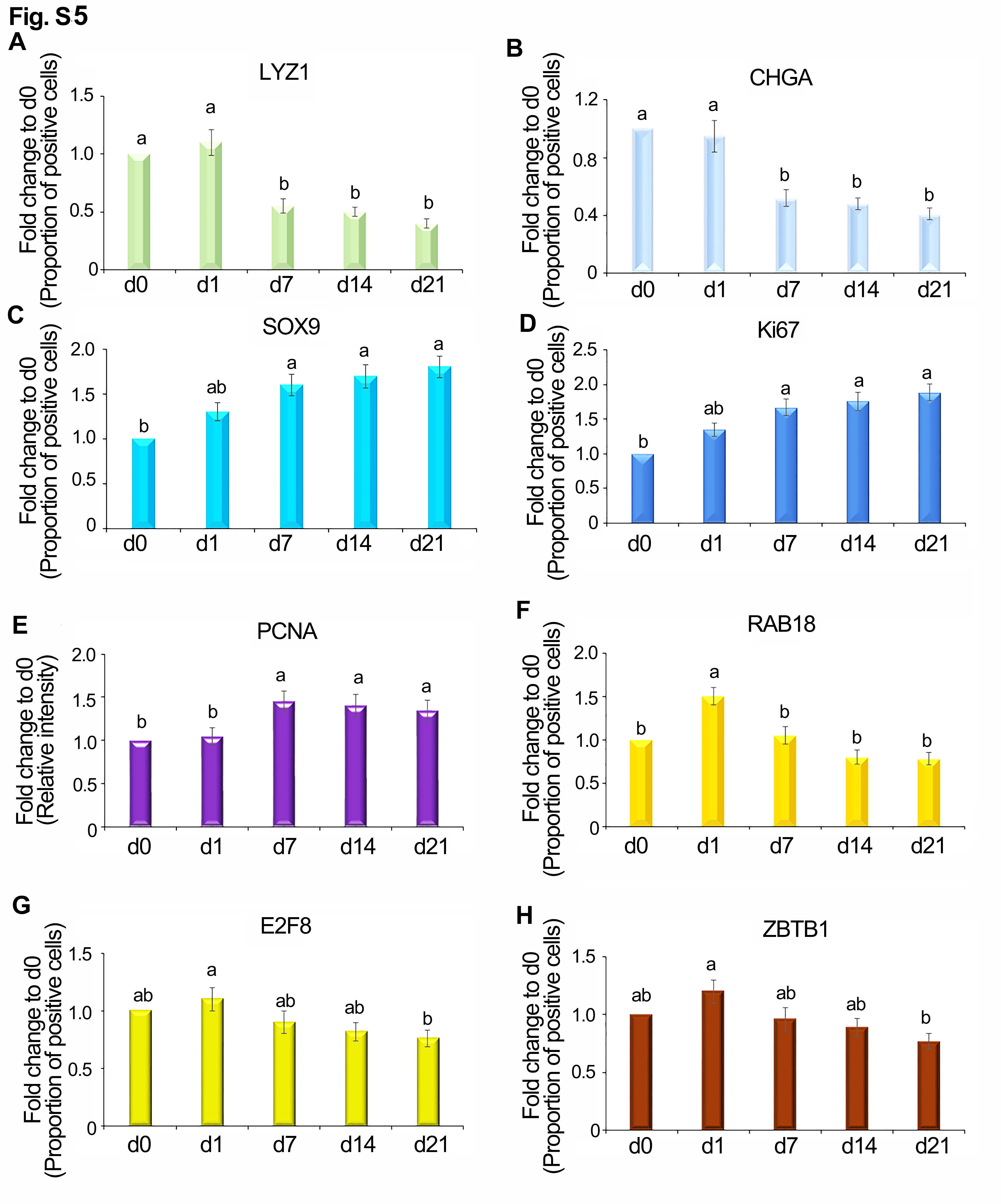

Supplement: Supplementary file 9 [file Image5.TIF]
