# Supplementary material for: Multi-Omics Uncover Neonatal Cecal Cell Development Potentials
Source: Front Cell Dev Biol. 2022 Jul 15;10:840298. doi: 10.3389/fcell.2022.840298 (PMC9334561; doi:10.3389/fcell.2022.840298)
Supplement: Supplementary file 4 [file DataSheet1.zip › Supplementary Table 1 Primary antibody informat.docx]

**Supplementary Table 25.** Primary antibodies

| **Gene symbol** | **Name** | **Cat. #** | **Predicted size** | **Source (Animal)** | **Company** |
| --- | --- | --- | --- | --- | --- |
| Actin | Rabbit anti-Actin antibody | D110001-0200 | 42kd | Rabbit (polyclonal) | BBI |
| PCNA | Rabbit Anti-PCNA antibody | ab18197 | 29kd | Rabbit (polyclonal) | Abcam |
| SOX9 | Rabbit Anti-Sox9 antibody | AB5535 | 65kd | Rabbit (polyclonal) | Merck Millipore |
| Vil1 | Rabbite anti-Villin antibody | ab130751 | 93kDa | Rabbit (polyclonal) | Abcam |
| FABP | Rabbit Anti-intestinal FABP antibody | bs-0898R | 28kd | Rabbit (polyclonal) | Abcam |
| CD3 | Rabbit Anti-cluster of differentiation 3 antibody | bs-10498R | 20kd | Rabbit (monoclonal) | Beijing Biosynthesis Biotechnology CO. |
| CCL5 | Rabbit Anti-CCL5/RANTES antibody | bs-20765R | 7.4/10kDa | Rabbit (polyclonal) | Beijing Biosynthesis Biotechnology CO. |
| E2F8 | Rabbit Anti-E2F8 antibody | bs-4265R | 94kd | Rabbit (polyclonal) | Beijing Biosynthesis Biotechnology CO. |
| APOA1 | Apolipoprotein A1 | bs-0849R | 28kDa | Rabbit (polyclonal) | Beijing Biosynthesis Biotechnology CO. |
| FTH1 | Rabbit Anti-Ferritin Heavy Chain/FTH1 antibody | bs-5907R | 20kd | Rabbit (polyclonal) | Beijing Biosynthesis Biotechnology CO. |
| Catenin | Rabbit Anti-delta 1 Catenin/CAS antibody | ab92514 | 108kd | Rabbit (monoclonal) | Abcam |
| MUC13 | Mouse Anti-MUC13 antibody | ab231159 | 55kd | Mouse (monoclonal) | Abcam |
| TFF3 | Rabbit Anti-Trefoil Factor 3 antibody | ab202967 | 9kd | Rabbit (polyclonal) | Abcam |
| IL-6 | Rabbit Anti-IL-6 antibody | bs-0782R | 23kd | Rabbit (polyclonal) | Beijing Biosynthesis Biotechnology CO. |
| CHGA | Rabbit Anti-Chromogranin A antibody | ab45179 | 86kd | Rabbit (polyclonal) | Abcam |
| LYZL1 | Rabbit Anti-LYZL1 antibody | bs-18593R | 15kd | Rabbit (polyclonal) | Beijing Biosynthesis Biotechnology CO. |
| RAB18 | Mouse Anti-RAB18 antibody | bsm-51333M | 23kd | Mouse (monoclonal) | Beijing Biosynthesis Biotechnology CO. |
| POU2AF1 (BOB1) | Rabbit Anti-BOB1 antibody | bs-1418R | 28kd | Rabbit (polyclonal) | Beijing Biosynthesis Biotechnology CO. |
| Ki67 | Rabbit Anti-ki67 antibody | Ab15580 | 358 | Rabbit (polyclonal) | Abcam |
| MYBL2 | Rabbit Anti-BOB1 antibody | bs-5960R | 77kd | Rabbit (polyclonal) | Beijing Biosynthesis Biotechnology CO. |
| GATA6 | Rabbit Gata binding factor 6 antibody | bs-1787R | 60kDa | Rabbit (polyclonal) | Beijing Biosynthesis Biotechnology CO. |
| ZBTB1 | Zinc finger and BTB domain containing 1 | bs-13563R | 82kDa | Rabbit (polyclonal) | Beijing Biosynthesis Biotechnology CO. |
